# Healthcare Organizational Resilience: A Systematic Review and Integrative Framework of Factors, Capabilities, Outcomes, and Context

**DOI:** 10.21203/rs.3.rs-11543209/v1

**Published:** 2026-10-30

**Authors:** Abdul-Hanan Saani Inusah, Atena Pasha, Xiaoming Li, Shan Qiao

**Affiliations:** University of South Carolina

**Keywords:** Organizational resilience, Healthcare organizations, Health services research, Resilience capabilities, Service continuity, Organizational recovery, Systematic review

## Abstract

**Background:**

Healthcare organizational resilience remains inconsistently defined and measured, with organizational factors, capabilities, and outcomes frequently conflated.

**Objective:**

To examine definitions and operationalizations, synthesize organizational factors, resilience capabilities, outcomes, and context, and develop an evidence-informed integrative framework.

**Methods:**

We conducted a systematic review following PRISMA 2020. We searched MEDLINE, PsycINFO, Web of Science, and Scopus from inception to 11 May 2026 for empirical studies examining healthcare organizations as resilient entities. We synthesized qualitative, quantitative, and mixed-methods evidence using a convergent integrated approach informed by Joanna Briggs Institute guidance. Inductively coded findings were classified by analytical role.

**Results:**

Forty-seven reports representing 45 studies (25 qualitative, 15 quantitative, five mixed-methods) across multiple countries were included; 31 examined COVID-19 or pandemic conditions, and most concerned hospitals. Operationalizations included direct measures, frameworks, qualitative inference, service proxies, and expert ratings. Six factor domains concerned leadership and governance; workforce and culture; information, communication, and digital technologies; preparedness and coordination; material, financial, and infrastructural resources; and external relationships and policy support. Five capabilities comprised anticipation and preparation, absorption, adaptation and reconfiguration, restoration and recovery, and learning and transformation. Four outcome groups were service continuity, safety and quality, recovery, and organizational viability. Immediate continuity sometimes depended on workforce overextension, deferred care, temporary support, or resource depletion. The framework distinguishes factors, capabilities, outcomes, and contextual conditions.

**Conclusions:**

Resilience assessment should extend beyond preparedness plans and immediate continuity to safety, workforce well-being, recovery, and longer-term viability. The hypothesis-generating framework requires prospective testing across healthcare settings and disruptions.

## 1.0 Background

Disruption is consequential in healthcare not merely because it changes demand, but because it can destabilize the organizational arrangements through which essential care is delivered. Acute shocks such as pandemics and disasters frequently intersect with chronic workforce shortages, supply instability, financial constraints, and sustained operational pressure, reducing organizational capacity precisely when demands on care are greatest [1-3]. Because resilience can be examined at multiple levels within healthcare, it is important to distinguish the healthcare organization as a resilient entity in its own right. In this sense, continuity depends not only on preparedness plans or available resources, but on whether organizations can coordinate decisions, mobilize and reconfigure resources, adapt established routines, and restore disrupted functions while protecting safety, quality, and longer-term viability [4-6]. These interrelated capacities are commonly encompassed by the concept of organizational resilience [7, 8].

Despite growing empirical attention, organizational resilience remains inconsistently defined and operationalized in healthcare research. Some studies assess resilience as a general organizational capacity, others focus on specific capabilities such as anticipation, absorption, adaptation, recovery, or learning, and others infer resilience from observable results such as service continuity, operational recovery, financial stability, or quality of care [6-8]. Consequently, organizational conditions such as leadership, staffing, information systems, and material resources may be considered alongside organizational capabilities such as anticipation and adaptation and outcomes such as continuity or recovery in conceptualizing organizational resilience. Because factors, capabilities, outcomes, and organizational conditions are frequently entangled within definitions and measures of organizational resilience, it is difficult to compare the evidence and determine what factors facilitate or impair resilient performance, what capabilities organizations require in response to disruption, and what outcomes indicate that resilient performance has been achieved or sustained.

Previous reviews have provided important but differently bounded syntheses of organizational resilience in healthcare. Hospital-focused reviews have identified factors relevant to preparedness, response, and recovery; synthesized organizational resources and capabilities; and examined how hospital resilience has been conceptualized, operationalized, and evaluated [4, 6, 9]. Reviews adopting a broader organizational perspective have focused principally on definitions and approaches to measurement or assessment, consistently identifying limited agreement about what constitutes organizational resilience and how it should be evaluated [7, 8]. Although some reviews have distinguished selected components, capabilities, phases, or outcomes, their different analytical roles have not been consistently separated within a single organization-level synthesis spanning healthcare organization types and qualitative, quantitative, and mixed-methods evidence. A synthesis that distinguishes how these elements function is therefore needed to clarify how empirical evidence fits together and provide a coherent conceptual basis for developing more consistent operational definitions and measures. We therefore conducted a systematic review of empirical studies in which a healthcare organization, including hospitals and other healthcare-provider organizations, constituted the resilient entity. The review aimed to characterize how healthcare organizational resilience was defined and operationalized; identify factors associated with resilient performance; synthesize the capabilities, outcomes, and contextual conditions reported across qualitative, quantitative, and mixed-methods evidence; and develop an evidence-informed, hypothesis-generating integrative framework of organizational resilience in healthcare. The central contribution of this review is an evidence-informed, hypothesis-generating framework that distinguishes organizational factors (what organizations have), resilience capabilities (what they do), outcomes (what they achieve), and contextual conditions that shape these relationships.

## 2.0 Methods

### 2.1 Study design

This systematic review was conducted and reported in accordance with the Preferred Reporting Items for Systematic Reviews and Meta-Analyses (PRISMA) guidelines [10]. Title and abstract screening, full-text screening, and data extraction were conducted by two independent reviewers. Disagreements were resolved through discussion and, when necessary, adjudication by a third reviewer.

### 2.2 Search strategy and information sources

A systematic literature search was conducted in MEDLINE (Ovid), PsycINFO (EBSCOhost), Web of Science Core Collection, and Scopus from database inception to May 11, 2026. The search strategy was developed by an experienced health sciences librarian, reviewed by all authors, and adapted as appropriate for each database. To maximize search sensitivity and minimize the likelihood of missing eligible studies, the search combined terms related to healthcare organizations with organizational resilience and conceptually related constructs, including adaptive capacity, absorptive capacity, transformative capacity, adjustment capacity, and related resilience terminology. The search strategy prioritized sensitivity over specificity and was intentionally not restricted to the exact phrase *organizational resilience*, recognizing that relevant empirical studies frequently describe organizational resilience using conceptually related terminology. The complete electronic search strategies for all databases are provided in *Appendix 1.*

### 2.3 Eligibility criteria

Eligibility criteria were specified a priori using a PICOS-informed framework. Studies were eligible for inclusion if they met all of the following criteria: (1) were original empirical studies using qualitative, quantitative, or mixed-methods designs; (2) examined the resilience of a healthcare organization, including hospitals, hospital systems, primary care organizations, community healthcare organizations, long-term care organizations, mental health organizations, rehabilitation organizations, or other healthcare-provider organizations; (3) investigated organizational resilience or an equivalent organizational construct, such as hospital resilience, adaptive capacity, resilient organizational functioning, organizational recovery, or service continuity, as the primary study objective, analytical framework, principal outcome, or central phenomenon; and (4) empirically examined at least one internal or external condition, resource, structure, relationship, or organizational arrangement in relation to organizational resilience or a resilience-related capability or outcome.

Because resilience may be examined at multiple levels within healthcare systems, this review specifically focused on studies in which the healthcare organization itself constituted the resilient entity. Studies were excluded when resilience was examined primarily at the level of individuals, teams, departments, discrete interventions, organizational functions, or health systems without organization-level findings.

No restrictions were placed on geographic location or type of eligible healthcare organization. Only peer-reviewed articles published in English were included. Reviews, editorials, commentaries, conference abstracts or papers, study protocols, conceptual or theoretical papers, and other non-empirical publications were excluded.

### 2.4 Study selection

All retrieved records were imported into Covidence for study management and screening. After duplicate removal, records underwent title and abstract screening according to the prespecified eligibility criteria. Reports considered potentially eligible underwent full-text review.

### 2.5 Data extraction and management

A standardized data-extraction matrix was used to collect bibliographic information; country and healthcare organization; study design and sample; context or disruption; the definition or conceptualization of organizational resilience; the measure or operationalization of resilience; organizational factors or themes examined; the reported role or direction of findings; and key resilience-related findings.

Complementary information was extracted from each eligible report, while overlapping samples and findings were counted only once in the cross-study synthesis.

### 2.6 Data synthesis and analysis

Data were synthesized using a convergent integrated approach to mixed-methods systematic reviews, informed by guidance from the Joanna Briggs Institute (JBI) [11]. Evidence was initially extracted in a form appropriate to each study design. Quantitative findings were translated into structured narrative statements describing the constructs or exposures examined, the outcomes assessed, the direction and magnitude of associations, and relevant contextual findings. Qualitative findings were summarized as themes, reported mechanisms, barriers, facilitators, organizational actions, observed results, and the context in which they were described. Findings were subsequently compared and integrated across study designs.

Initial coding was inductive: codes were generated from the extracted findings rather than imposed from a pre-existing thematic framework. Coding was iterative and involved comparing conceptually similar findings within and across studies. Two reviewers coded the findings and refined the developing categories through comparison and consensus; unresolved disagreements were adjudicated by a third reviewer. Synonymous or closely related codes were consolidated only when their meanings and organizational levels were comparable. The resulting codes were grouped into broader thematic categories.

For the descriptive synthesis of measurement and operationalization, each unique study was assigned one primary category: direct or model-based assessment; named framework or index; qualitative inference; performance or service proxy; or expert rating. These categories were made mutually exclusive at the study level to prevent double counting, while hybrid features were retained in detailed extraction.

### 2.7 Classification of analytical roles

To distinguish conceptually different roles within the organizational-resilience literature, each specific finding was subsequently classified according to its primary analytical role as a factor, capability, outcome, or contextual condition.

A *factor* was defined as a proximal internal or external condition, resource, structure, relationship, or organizational arrangement that could enable or constrain resilient performance, such as staffing capacity, leadership and decision arrangements, information systems, material resources, or interorganizational support. A *capability* described an organizational ability or enacted response through which the organization anticipated or prepared for disruption, absorbed its effects, adapted or reconfigured its functioning, restored or recovered disrupted functions, or learned and transformed in response to experience. An *outcome* described an observable organizational or service-delivery result that was maintained, restored, or achieved during or following disruption. A *contextual condition* described a background characteristic of the organization, setting, or disruption that could shape how factors, capabilities, or outcomes operated or were interpreted, rather than functioning as a resilience factor in itself. These included organization type and ownership, resource setting and geography, service complexity, disruption type, severity and duration, and the broader policy and regulatory environment.

Because the same concept could occupy different analytical roles across studies, classification was determined by how the construct functioned in the reported finding. For example, staffing availability was classified as a factor when it represented existing workforce capacity, whereas staff redeployment was classified as a capability because it represented an organizational response to disruption. Continued delivery of essential services was classified as an outcome. Similarly, recovery was classified as a capability when it referred to an organizational ability or restorative process and as an outcome when it referred to the measured extent or timeliness with which disrupted activity or functioning was restored.

An individual study could contribute separate findings to more than one analytical role. Where a study combined factors, capabilities, or outcomes within a composite resilience measure or framework, the underlying components were classified according to their represented analytical roles for the integrated synthesis while the original operationalization was retained in the descriptive synthesis of measurement.

The resulting factor domains, capabilities, outcome groups, and contextual conditions were synthesized into an evidence-informed, hypothesis-generating integrative framework. The framework was intended to organize recurring cross-study patterns and proposed relationships rather than represent a validated causal model.

## 3.0 Results

### 3.1 Study selection

Database searching identified 14,995 records: 4,583 from Scopus, 4,347 from PsycINFO, 3,471 from Web of Science, and 2,594 from MEDLINE. After removal of 5,891 duplicate records, 9,104 records underwent title and abstract screening, of which 8,816 were excluded. Full texts of 288 reports were assessed, and 241 were excluded (Fig. 1). The remaining 47 eligible reports represented 45 unique studies because two study families each contributed two publications [12-15].

### 3.2 Characteristics of the included studies

Reports were published between 2017 and 2026. Of the 45 studies, 25 contributed qualitative evidence, 15 quantitative evidence, and five used mixed-methods designs. Study designs ranged from single-organization case studies to multi-organization surveys and administrative-data cohorts. Detailed study characteristics are presented in Table 1.

The evidence base was geographically diverse but predominantly hospital-centered. It included public, private, nonprofit, academic, teaching, rural, tertiary, community, and long-term care hospitals, as well as hospital networks and a small number of other healthcare-provider organizations. Distinct organizational settings included a nationwide virtual-care staffing program [16], organizations providing HIV services [17], and long-term care hospitals [18]. Studies were conducted in high-, middle-, and lower-resource settings and included single-country, multinational, and regional investigations.

Thirty-one studies examined COVID-19 or pandemic-associated conditions. The remaining studies broadened the evidence beyond acute pandemic response by examining service continuity and recovery after Super Typhoon Haiyan [19], the Nepal earthquake [20], and Hurricane Harvey [21]; leadership across disaster and climate-event cycles [22]; prolonged austerity [23]; chronically high operational pressure [24]; technological and operational disruption [25]; and routine or general organizational-resilience conditions [26-29].

### 3.3 Definition and conceptualization of organizational resilience

Organizational resilience was not treated as a single, uniform construct. Most studies described it as an organizational capacity or capability to prepare for, absorb, respond to, adapt to, and recover from disruption while maintaining essential functions. A second, overlapping orientation treated resilience as a dynamic or phase-based process in which anticipation, coping, adaptation, learning, and transformation unfolded over time [30-33]. A third orientation emphasized observable continuity, recovery, or organizational performance rather than a general latent resilience construct [34-38]. Several studies did not provide a standalone definition; in these cases, the meaning of resilience was inferred from the organizational phenomenon, analytical framework, or outcome examined [39-41]. Across these orientations, factors, capabilities, and outcomes were seldom explicitly distinguished. Conditions such as leadership, staffing, and resources; capabilities such as anticipation and adaptation; and outcomes such as continuity and recovery were frequently incorporated within the same definition, framework, or measure of organizational resilience.

### 3.4 Measurement and operationalization of organizational resilience

Organizational resilience was operationalized using direct or model-based assessments (11 studies), named frameworks or indices (17), qualitative inference (10), performance or service proxies (six), and expert ratings (one). Direct assessments included organizational-resilience scales, the Benchmark Resilience Tool, and latent-variable models [13, 15, 28, 42]; framework approaches included the Hospital Resilience Index, Resilience Analysis Grid, disaster-cycle and capability models, and HoSPiCOVID [18, 19, 43-45].

Proxy outcomes included elective-activity recovery in England and Italy [34, 35], hospital margins [36], service quality [37], disruption mitigation and resource reconfiguration [25], and patient survival under caseload pressure [38]. Detailed operationalizations appear in Table 1.

### 3.5 Factors associated with organizational resilience

Study-level qualitative and quantitative findings are presented in Table 2A and 2B, respectively, while Table 3 summarizes the integrated evidence across domains. The convergent synthesis produced six interrelated factor domains: (1) *leadership, governance, and decision authority;* (2) *workforce resources, well-being, and organizational culture;* (3) *information systems, communication and digital technologies;* (4) *preparedness arrangements, organizational structures, and coordination arrangements;* (5) *material, financial, and infrastructural resources;* and (6) *external networks, community relationships, and policy support*. Rather than operating independently, these factors commonly shaped the conditions under which organizations could anticipate, absorb, adapt to, recover from, or learn from disruption.

#### 3.5.1 Leadership, governance, and decision authority

Clear responsibility, timely leadership, multidisciplinary crisis structures, and links between strategic and frontline decision-making helped organizations interpret changing conditions and respond during disruption [21, 22, 30, 46]. The important distinction was not centralization versus decentralization alone, but the combination of shared priorities with delegated operational authority [47, 48]. Participatory leadership, staff involvement, trust, and open communication supported workable local responses [23, 49].

Conversely, contradictory directives, ambiguous authority, and poorly integrated formal and informal leadership delayed decisions, weakened trust and documentation, and encouraged local workarounds that were useful immediately but difficult to institutionalize [40, 41]. Traditional management approaches, limited leadership capability, politicization, and resistance to change were also identified as barriers [50, 51].

Survey findings showed that governance-related associations were not uniform. Self-organization was strongly associated with adaptive capacity in one hospital survey, whereas responsibility was not significantly associated with planning [26]. Resilient leadership was associated with organizational resilience directly and indirectly through nurses' well-being [28]. Leadership was also positively associated with public-hospital resilience, partly through risk-control capability [52]. In an Indonesian structural model, strategic leadership was positively associated with resource reconfiguration and hospital sustainability but negatively associated with network capability [42]. Together, these findings point to governance as a configuration of clear authority, participation, delegation, and coordination rather than a standalone leadership attribute.

#### 3.5.2 Workforce resources, well-being, and organizational culture

Workforce availability, clinical and operational expertise, cross-training arrangements, and staff support shaped how organizations responded to disruption. These resources facilitated staff redeployment, role expansion, work redesign, and coordinated service delivery [16, 32, 43, 53]. Organizational cultures characterized by trust, openness to change, collective problem-solving, and staff involvement supported coordinated action [23, 49]. Staff safety, psychological support, recognition, and meaningful work were also described as conditions for sustaining organizational effort during prolonged disruption [17, 20, 24].

However, workforce responses that maintained immediate service continuity could also increase longer-term vulnerability. Redeployment, expanded roles, and improvisation helped organizations absorb and adapt to disruption, but accumulated illness, fatigue, anxiety, staffing shortages, and unfamiliar roles increasingly constrained these responses as crises persisted [18, 30, 54, 55]. Improvisation was often effective during the acute phase but became exhausting when it was not supported by clear plans, opportunities for recovery, and additional staffing capacity [48]. This distinction is important: maintaining services in the short term did not necessarily indicate a sustainable workforce capability.

Quantitative findings further distinguished direct and indirect relationships. Strategic human-resource management was positively associated with cognitive, behavioral, and contextual resilience [27]. Occupational health and safety was associated with hospital resilience directly and indirectly through staff engagement and staff resilience [29], while nurses' well-being partially mediated the association between resilient leadership and organizational resilience [28]. Greater medical-staffing intensity was associated with faster recovery of elective activity in Italy [35]. In contrast, staff and digital skills were not directly associated with hospital resilience in one model, with their relationship operating through external information integration [14]. These findings suggest that workforce-related factors operated through staffing capacity, supportive management and well-being arrangements, and their integration with broader organizational systems.

#### 3.5.3 Information systems, communication and digital technologies

Timely, credible, and bidirectional communication supported shared situational awareness, coordination, and rapid adjustment. Combining organization-wide updates with targeted team communication and frontline feedback helped coordinate responses [32, 44] while central data was more useful alongside local knowledge and professional experience [24]. Conversely, fragmented, contradictory, delayed, excessive, or poorly documented information impeded coordination and organizational learning [21, 40, 56]. Thus, information quality, timing, and flow mattered alongside its availability.

Digital tools supported telehealth, remote coordination, surveillance, supply and device tracking, and clinical information exchange, but their contribution depended on integration into organizational processes. Existing virtual-care expertise facilitated service continuity in a nationwide contingency staffing program [16], whereas inadequate telehealth infrastructure and implementation constrained adaptation in some HIV service organizations [17]. Healthcare 4.0 applications were described as supporting monitoring, anticipation, response, and learning, with contributions varying by implementation maturity and ownership context [57]. Expert ratings likewise distinguished technologies with broad perceived contributions from those with more capability-specific contributions [58].

Quantitative associations were mixed. Digital technologies and external information integration were positively associated with hospital resilience, whereas staff skills and internal information integration were not directly associated; staff skills operated indirectly through external information integration [14]. In the linked analysis, service complexity and operational efficiency changed the strength or direction of several relationships [15]. Digital-technology innovation and synergy were positively associated with resilience, while compatibility and security were not statistically supported [59]. Artificial intelligence (AI) integration within knowledge-management systems was associated with less operational disruption and greater resource reconfiguration; technical complexity weakened disruption mitigation, while trust strengthened reconfiguration [25]. Customer-relationship-management resources were associated with resilience indirectly through organizational capabilities and risk and crisis management [13].

#### 3.5.4 Preparedness arrangements, organizational structures, and coordination arrangements

Preparedness plans, protocols, incident-command structures, simulations, and prior disaster experience provided a foundation for organizational response. However, arrangements designed for short, bounded emergencies were often inadequate for prolonged, evolving disruptions [18, 47, 55]. Plans were more useful when familiar, integrated into routine practice, activated promptly, and flexible enough to accommodate changes in roles, patient flows, capacity, and decision structures [19, 21, 31, 46].

Formal structures supported accountability and coordination, while informal networks and frontline improvisation enabled rapid adjustment. Arrangements combining clear authority with local input and lateral coordination supported responses to changing demands, particularly when effective emergent practices were subsequently incorporated into formal structures [41, 48]. Under chronic operational pressure, regular multidisciplinary meetings combined centralized data with local knowledge to identify risks and allocate responsibility [24].

Importantly, the presence of preparedness arrangements did not necessarily indicate operational readiness. Both formal and informal emergency-response practices were associated with higher self-reported hospital resilience, with consistently larger associations for informal practices [39]. Information and communication; teams, equipment, and tools; standard operating procedures; training; and the built environment were identified as interdependent resources rather than independent contributors [43]. In long-term care hospitals, business-continuity plans were commonly reported, but implementation was often considered insufficient, and infectious-disease response teams were frequently established only after the pandemic began [18]. These findings distinguish having formal plans from the organizational capacity to implement and adjust them during disruption.

#### 3.5.5 Material, financial, and infrastructural resources

Physical space, equipment, protective supplies, oxygen, medicines, information infrastructure, and flexible funding provided a material foundation for maintaining and adapting services. Shortages constrained organizational responses even where leadership and staff commitment were strong [30, 37, 54, 56]. Substitutable spaces, adaptable isolation capacity, backup infrastructure, and rapid access to external supplies expanded response options [20, 21]. However, following Super Typhoon Haiyan, both hospitals initially maintained emergency services, but their longer-term trajectories diverged. One restored operations, while the other ceased inpatient services after exhausting critical financial, material, and workforce resources [19].

Quantitative findings further illustrated the limitations of individual resources. In England, most measured hospital supply characteristics were not systematically associated with elective-activity recovery [34]. Federal relief funds improved total margins among financially strained and vulnerable rural hospitals in the United States, but the benefits diminished as support receded and pre-existing fragility persisted [36]. Similarly, more advanced hospital infrastructure did not significantly modify the adverse association between COVID-19 caseload pressure and patient survival [38].

Together, these findings suggest that material and financial resources expanded organizational response options but were not independently sufficient to ensure recovery or sustained resilience. Their contribution depended on availability, timely replenishment, and the ability to mobilize and reconfigure resources as demands changed.

#### 3.5.6 External networks, community relationships, and policy support

Interorganizational partnerships, regional coordination, government and public-health support, community relationships, and supply-sharing arrangements expanded organizational access to information, staff, services, and material resources during disruption [16, 17, 19, 37]. Regional evidence further emphasized the importance of community connections and coordinated technical and logistical support from ministries of health and international organizations for service continuity and recovery [33]. Conversely, weak cross-sector cooperation, fragmented external communication, and misaligned regional or national directives constrained organizational responses [18, 51].

Quantitative findings showed that external relationships were not uniformly associated with resilience. External information integration was positively associated with hospital resilience and mediated the relationships between other organizational resources and resilience in Italy [14]. In Indonesia, perceived government regulation was positively associated with strategic leadership, whereas network capability had negative relationships with strategic leadership and resource reconfiguration [42]. These findings suggest that the contribution of external relationships depended on the nature and practical usefulness of the support they provided, rather than their presence alone.

### 3.6 Cross-cutting resilience capabilities

Across the synthesized evidence, five higher-order capabilities were identified: i) *anticipating and preparing*; ii) *absorbing disruption*; iii) *adapting and reconfiguring;* iv) *restoring and recovering;* and v) *learning and transforming*.

#### 3.6.1 Anticipate and prepare

Anticipation involved monitoring threats and organizational capacity, scenario planning, exercises, risk control, clear escalation arrangements, and using lessons from previous events to guide preparedness. Preparedness was stronger when these activities were embedded in routine organizational practice and linked to current staffing, supply, and communication systems [19, 22, 56]. Across several studies, however, formal plans coexisted with low operational readiness, weak anticipation, or plans that were poorly suited to prolonged or changing disruptions [18, 43, 55].

#### 3.6.2 Absorb disruption

Absorptive capability involved mobilizing reserve capacity, redeploying staff, separating patient flows, substituting spaces and roles, obtaining external support, and temporarily reprioritizing services [16, 20, 30]. These strategies helped maintain immediate continuity, but the response was sometimes accompanied by deferred services, reliance on temporary financial support, resource depletion, and workforce overextension. Absorption could therefore be achieved without full recovery or sustainable organizational resilience.

#### 3.6.3 Adapt and reconfigure

Adaptation was widely demonstrated across the included studies. Organizations altered governance, roles, staffing, capacity, protocols, digital delivery, supply arrangements, and care pathways in response to changing demands [31, 43, 44, 46]. Successful reconfiguration depended on timely information, clearly defined frontline decision authority, coordination, and access to resources. Improvisation by individual teams was less sustainable when organizational structures did not recognize, coordinate, or support the changes introduced [40, 48].

#### 3.6.4 Restore and recover

Recovery capability involved rebuilding operational capacity, restoring disrupted functions, addressing deferred demand, and moving from temporary crisis arrangements toward stable service delivery. Reported recovery processes included remobilizing staff and resources, resuming routine services, using external partnerships, and restoring damaged infrastructure and operational capacity [19, 20, 31].

#### 3.6.5 Learn and transform

Learning involved reflection, documentation, feedback, revision of plans and routines, and incorporation of effective emergent practices into routine organizational processes. Studies reported learning between crisis phases and some formalization of locally developed arrangements, but also the loss of improvements achieved early in the response, incomplete documentation, and difficulty converting improvisation into lasting organizational change [40, 41, 47, 55]. Transformation was commonly included in definitions and frameworks but was less often directly demonstrated or measured than immediate adaptation.

### 3.7 Outcomes of organizational resilience

Four recurring outcome groups were identified: i) *continuity of essential services and operations*; ii) *safety and quality of care*; iii) *extent and timeliness of recovery*; and iv) *organizational sustainability and viability*. These outcomes differed substantially in their level, operationalization and period of observation and were therefore not directly comparable across studies.

Continuity was widely described across the included studies. Reported outcomes included maintenance of outpatient, inpatient, triage, and virtual services; continuity of HIV and other essential services; maintenance of patient flow; and continued emergency care following major disruption [16-19]. However, continuity of selected services did not necessarily indicate that the organization remained fully functional. In some cases, emergency or crisis-related services were maintained by suspending elective or routine care, requiring staff to work beyond normal limits, or relying on temporary resources [20, 31, 55].

Safety and quality were examined less consistently and through heterogeneous indicators. Evidence included the perceived maintenance of hospital-service quality during COVID-19, patient survival under increasing caseload pressure, and the reported effects of disaster response on routine activity and quality of care [20, 37, 38].

Recovery outcomes concerned the extent or speed with which disrupted activity or organizational functioning was restored. Elective-activity recovery was measured relative to pre-pandemic baselines in England and Italy, while longer-term recovery following Super Typhoon Haiyan differed markedly between two hospitals: one restored normal or near-normal operations within weeks, whereas the other subsequently ceased inpatient operations after exhausting critical resources [19, 34, 35].

Organizational sustainability and viability were represented through financial performance, continued organizational functioning, and the ability to sustain operations beyond the immediate response. Evidence included changes in rural-hospital operating margins and the temporary stabilizing contribution of Provider Relief Funds, hospital sustainability as an explicit outcome of leadership and resource reconfiguration, and the longer-term consequences of resource exhaustion following an initially successful emergency response [19, 36, 42]. These findings therefore illustrated that apparent short-term resilience, particularly continued service delivery, could coexist with longer-term organizational vulnerability when continuity depended on workforce overextension, deferred care, temporary financial support, or depletion of critical resources.

### 3.8 Contextual conditions and variation

Reported relationships between organizational factors and resilience varied according to organization type, ownership, resource setting, service complexity, geography, disruption type and duration, and the policy and regulatory environment. Ownership and hospital type were associated with different resilience or recovery patterns across studies; however, the evidence did not support a consistent advantage for any particular ownership or hospital type [19, 26, 35, 57]. Regional variation in elective-activity recovery remained after adjustment for measured hospital and catchment characteristics [34].

Duration emerged as an important contextual condition. During COVID-19, arrangements that supported rapid action in the acute phase became more difficult to sustain as ad hoc organizing became exhausting, cohesion and proactiveness declined, resource constraints became more prominent, and hospitals struggled to manage crisis-related and routine care simultaneously [48, 55]. Catastrophe plans designed for bounded emergencies also required continuing adjustment as the pandemic evolved [47]. Following Super Typhoon Haiyan, initial emergency-service continuity similarly did not ensure longer-term functioning when supplies, financing, workforce capacity, and external support could not be sustained [19].

### 3.9 Evidence-informed integrative framework

The synthesis informed the integrative framework presented in Fig. 2, in which six factor domains may support five interrelated resilience capabilities. These capabilities may contribute to four outcome domains: continuity of essential services and operations; safety and quality of care; extent and timeliness of recovery; and organizational sustainability and viability. Contextual conditions may shape each component and the relationships among them, while feedback from outcomes may inform subsequent organizational learning and adaptation.

The framework is evidence-informed and hypothesis-generating. It organizes recurring cross-study patterns and proposed relationships; it does not assign statistical-significance labels, imply that every factor was examined in every setting, or establish causal ordering. Its principal contribution is to distinguish factors associated with resilience from the capabilities through which organizations enact resilient performance and the outcomes through which that performance is observed, while locating these relationships within their contextual conditions

## 4.0 Discussion

### 4.1 Principal findings

This review identified six factor domains spanning leadership and governance, workforce resources and culture, information and digital technologies, preparedness and coordination, material and financial resources, and external relationships and policy support. Five resilience capabilities, anticipating, absorbing, adapting, recovering, and learning, were distinguished from four outcome groups: service continuity, safety and quality, recovery, and organizational viability. The relevance of these elements varied across organizational, resource, policy, and disruption conditions. Short-term service continuity did not always indicate longer-term organizational viability [19].

These factor domains broadly align with those identified in previous reviews [4, 6, 9, 60]. Against the conceptual and measurement heterogeneity documented in earlier syntheses [7, 8, 61], this review's contribution is to distinguish the analytical roles of recurring resilience-related elements rather than introduce entirely new domains.

### 4.2 Theoretical contribution of the integrative framework

Previous reviews differentiated selected resilience resources, capabilities, phases, and outcomes, but these roles have not been consistently separated within a single organization-level synthesis across healthcare settings [4, 6, 9]. The integrative framework addresses this gap by clarifying how these elements may relate without treating them as interchangeable.

This distinction matters because the presence of particular resources or the achievement of a favorable outcome does not alone demonstrate organizational resilience. Factors may enable or constrain capabilities through which organizations anticipate, absorb, adapt to, recover from, and learn from disruption; outcomes represent what is maintained, restored, or achieved. Contextual conditions may shape these relationships, while feedback from outcomes may inform subsequent learning and adaptation. The framework therefore treats resilience as a dynamic phenomenon and provides a basis for distinguishing enabling conditions, enacted responses, and observed performance in future assessment.

These relationships remain hypothesis-generating and require prospective testing; the review does not establish causal pathways.

### 4.3 Limitations and challenges in the existing evidence

Consistent with previous reviews, organizational resilience was inconsistently defined and measured [6-8]. Some studies combined organizational conditions, such as leadership and staffing, with capabilities, such as anticipation and adaptation, and outcomes, such as continuity or recovery, within the same definition or measure. This conflation obscured whether studies assessed enabling conditions, organizational responses, or observed performance. Operationalizations also varied from direct or model-based assessments and named frameworks to qualitative inference and performance proxies, limiting cross-study comparison.

Much of the quantitative evidence was cross-sectional, self-reported, or based on proxy outcomes. Qualitative studies illuminated organizational processes and context but were not designed to estimate the relative importance of individual factors. These designs support identification of recurring patterns but provide limited grounds for establishing causal ordering or how relationships evolve over time.

The evidence was predominantly hospital-based, and 31 of the 45 studies concerned COVID-19 or pandemic-associated conditions. Although informative about prolonged crisis response, this concentration leaves limited evidence from primary care, community and mental-health organizations, long-term care, other provider settings, routine operational pressure, and non-pandemic disruptions. Earlier hospital-focused reviews similarly highlight the field's emphasis on hospitals and disasters [4, 6, 9].

### 4.4 Recommendations for future research

Future studies should define the resilient entity, distinguish organizational factors, capabilities, outcomes, and contextual conditions, and specify whether resilience is measured directly or inferred from service continuity, recovery, financial performance, or quality-of-care proxies.

More comparable organization-level measures should be developed and validated across healthcare settings, including content validity, reliability, and construct validity. Where feasible, such measures should be combined with objective service or administrative indicators rather than relying on a single metric.

Longitudinal, comparative, and multilevel designs could examine how factors relate to capabilities and outcomes over time, and how these relationships vary by organization type, resource setting, and disruption type or duration. Research should also extend to primary care, community and mental-health organizations, long-term care, and other provider settings beyond hospitals and pandemics.

### 4.5 Strengths and limitations of the review

This review integrated qualitative, quantitative, and mixed-methods evidence using explicit organization-level eligibility criteria and linked reports from the same underlying studies. By characterizing definitions and measures and classifying findings according to analytical role, it developed a cross-study, evidence-informed framework.

Limitations include restricting inclusion to English-language, peer-reviewed studies and substantial heterogeneity that precluded meta-analysis. Including related organizational-resilience constructs and proxy outcomes broadened the synthesis but necessitated interpretive classification. Despite broad search terms, studies of organizational adaptation, crisis management, surge capacity, or business continuity without resilience-related terminology may have been missed. As original studies did not always distinguish analytical roles, some coding decisions depended on how constructs functioned in the reported findings.

### 4.6 Implications for health services, policy, and public health practice

The findings have implications for how organizational resilience is assessed and supported. Preparedness plans and short-term service continuity alone may be insufficient indicators of resilience. Some organizations maintained services through extraordinary workforce effort and improvisation [48, 55], while initial continuity could coexist with subsequent service disruption when financial, material, and workforce resources were exhausted [19]. Resilience assessment should therefore consider not only service continuity, but also safety and quality, workforce well-being, the extent and timeliness of recovery, and longer-term organizational viability. Monitoring these dimensions together may help identify vulnerabilities obscured by favorable short-term performance.

For policymakers and healthcare leaders, the synthesis suggests the importance of combining clear strategic direction with operational flexibility, embedding preparedness in routine practice, strengthening information systems, protecting workforce capacity, and maintaining dependable resources and external partnerships [1, 21, 33, 44]. Beyond the presence of these arrangements, preparedness and resource-planning decisions should consider whether organizations can mobilize and adapt them as demands change, particularly during prolonged disruption. Their relevance may vary by organization type, resource setting, disruption severity and duration, and the wider policy environment.

At the health-system level, resilience planning should also consider whether maintaining one service involves deferring or displacing care elsewhere in the delivery network. This emphasizes the importance of coordination across organizations and attention to the recovery of disrupted services. The proposed framework may therefore provide a structure for context-sensitive assessment, planning, and priority-setting rather than a validated policy instrument or universal checklist.

## 5.0 Conclusion

This review distinguishes organizational factors, resilience capabilities, outcomes, and contextual conditions within an evidence-informed, hypothesis-generating framework rather than a validated causal model. Future studies should test these relationships using validated organization-level measures across healthcare settings and disruptions. For policymakers, resilience assessment should extend beyond preparedness plans and immediate service continuity to safety and quality, workforce well-being, recovery, and longer-term organizational viability.

## Supplementary Material

This is a list of supplementary files associated with this preprint. Click to download.

• Appendix.docx

## Figures and Tables

**Figure 1:**
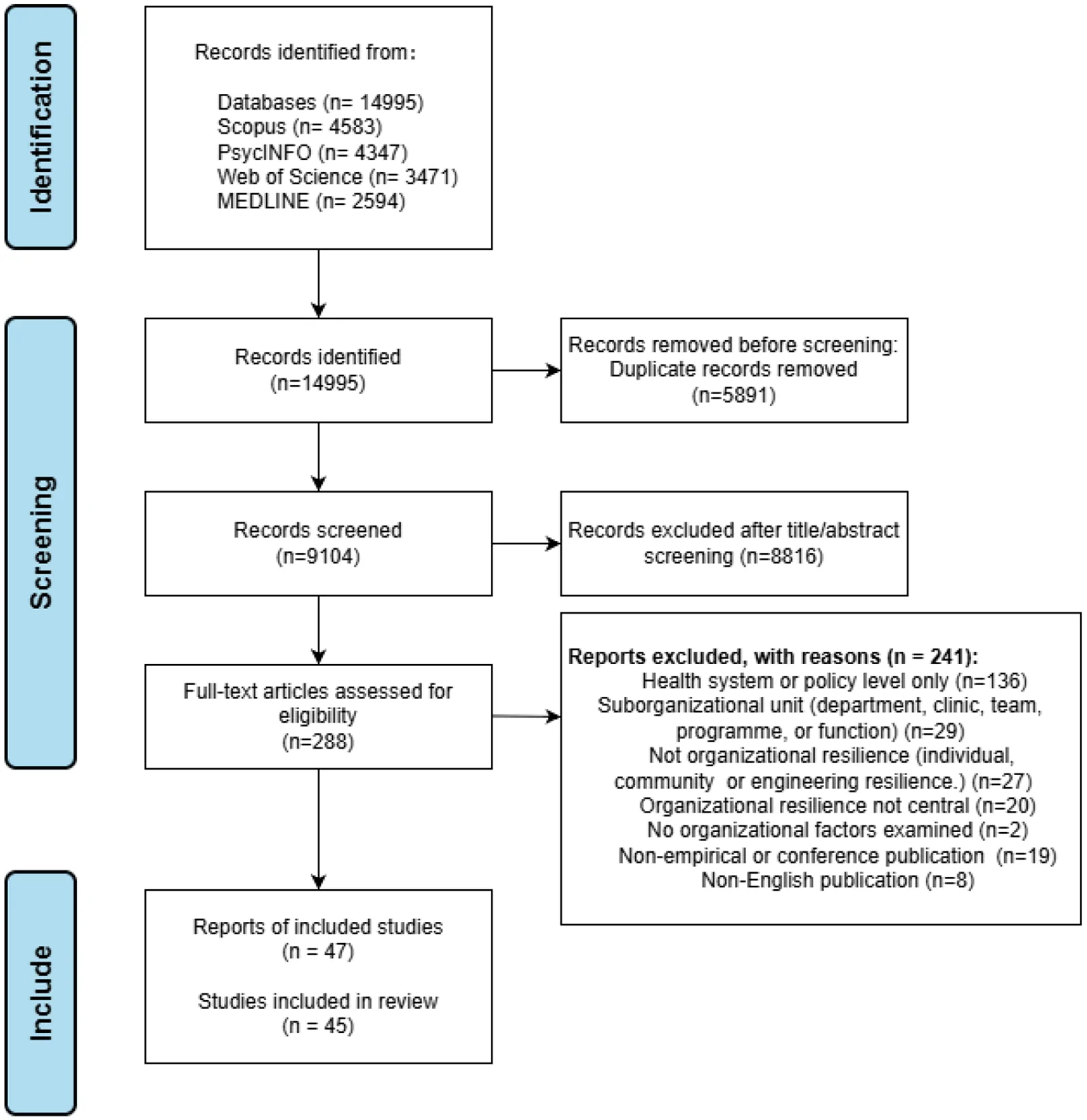
PRISMA 2020 flow diagram of the study selection process.

**Figure 2:**
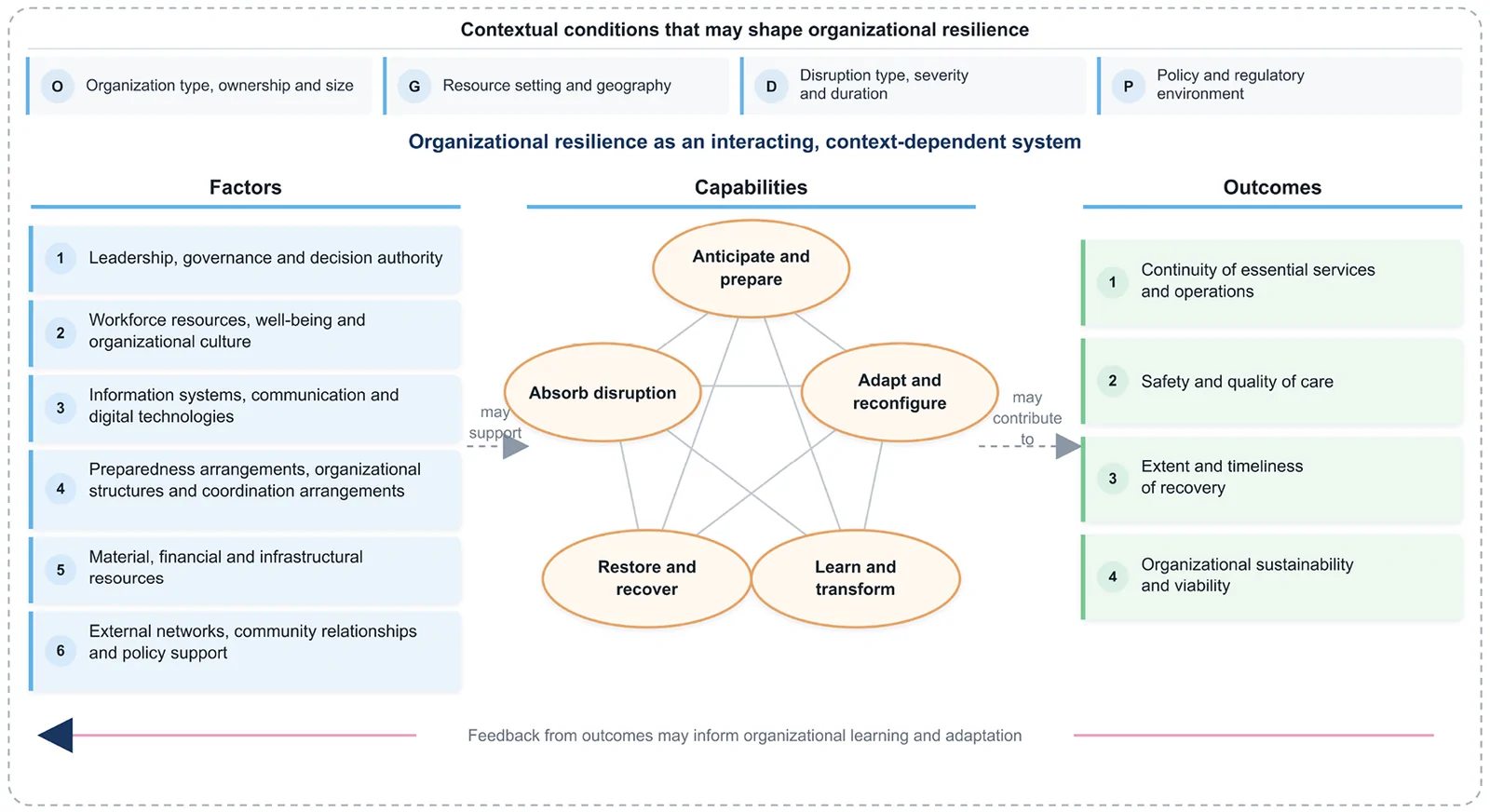
Evidence-informed integrative framework of organizational resilience in healthcare organizations.

**Table 1:** Characteristics and operationalization of organizational resilience in included studies

Study	Country and healthcare organization	Study design and sample	Context/disruption	Definition/conceptualization of organizational resilience	Measure/operationalization
Afsar 2026	Türkiye; university, Ministry of Health (state, training and research, and city), and private hospitals in Istanbul	Cross-sectional and descriptive survey; stratified random sample of 1,143 hospital employees across clinical, allied health, technical/biomedical, and administrative groups	General/Routine assessment of hospital resilience	The ability to maintain essential functions during disruptions, recover efficiently, and learn and adapt through experience	13-item Turkish-validated Organizational Resilience Scale adapted from Whitman et al.; adaptive capacity and planning subscales; summed scores, with higher scores indicating greater resilience.
Al-Ayed 2019	Country not explicitly reported; private hospitals	Quantitative questionnaire survey, 500 randomly selected administrative staff from private hospitals, with 449 returned questionnaires	General/Routine organizational resilience	The capacity to respond appropriately to a situation and make changes that reduce the effects of unexpected threats; conceptualized as cognitive, behavioral, and contextual resilience	Organizational resilience was operationalized through cognitive, behavioral, and contextual dimensions, with three indicators per dimension in the analyzed measurement model (9 indicators total), adapted from Lengnick-Hall et al.; five-point Likert scale.
**Atkinson 2024**	United States; nine rural and urban hospitals	Cross-sectional online survey with confirmatory factor analysis, quantile regression, and linear regression; purposive sample of 379 clinical and nonclinical hospital staff	During the COVID-19 pandemic	No standalone formal definition reported; treated as a self-reported organizational outcome of hospital emergency response during crisis	Five-item self-reported hospital resilience measure adapted from previously validated scales and averaged into an equally weighted score; HERO-36 separately assessed 36 formal and informal emergency-response practice items across four formal and four informal domains.
**Bosque-Mercader 2025**	England; National Health Service acute hospital trusts with type 1 emergency departments	Quantitative panel study using Hospital Episode Statistics; 97–121 trusts and 1,130-1,452 hospital-month observations	After COVID-19	The capacity to recover regular activity levels after a shock to the health system, assessed through recovery of elective activity in 2021 relative to 2019	Monthly ratio of elective activity in 2021 to the corresponding month in 2019, log-transformed for regression analysis; assessed for total, surgical, non-surgical, and specialty-specific activity.
**Cannedy 2022**	United States; nationwide Veterans Health Administration (VHA) Clinical Resource Hub program operating across 18 regional networks	Qualitative formative evaluation using semi-structured telephone interviews and rapid analysis; 26 stakeholders comprising 18 regional hub directors representing 17 VHA networks and eight national program leaders	During COVID-19	The capacity of health actors and institutions to respond effectively to and recover from crises while maintaining core functions; findings were interpreted through adaptiveness, self-regulation, and integration, with staff well-being proposed as an additional resilience component	Resilience was inferred qualitatively from themes concerning CRH pandemic-response actions, barriers, facilitators, and partnerships identified through rapid analysis of semi-structured interviews.
**Cuno-Chunga 2025**	Peru; public hospital in Abancay, Apurimac	Qualitative exploratory case study using semi-structured interviews and thematic analysis; convenience sample of 10 contracted hospital employees	Resource-constrained setting	A process and an outcome and examined through adaptation to organizational change, emotional management in the workplace, and innovation in organizational processes	Expert-validated semi-structured interview guide organized around adaptation, emotional management, and organizational innovation; thematic and semantic analysis.
de Almeida Marques 2026	Portugal; three public hospitals in Northern Portugal (CHVNGE, Hospital de Santo Antonio, and CMIN)	Qualitative multiple-case study; 41 semi-structured interviews with managers, department heads, clinical coordinators, and frontline leaders, supplemented by organizational and public documentary sources	During COVID-19	No standalone formal definition reported; resilience was implicitly conceptualized as the capacity to sustain operational continuity, adapt, and learn through the interaction of hierarchical governance, informal leadership, frontline autonomy, lateral coordination, and collective sensemaking	Resilience was inferred through deductive thematic analysis of interviews and documentary sources, supported by triangulation and reconstruction of three critical governance incidents involving authority conflict, coordination, adaptation, and organizational learning
**de Araujo Oliveira 2023**	Brazil; 400-bed public university referral hospital for infectious diseases in Pernambuco	Qualitative evaluative single-case study using data triangulation; 16 documents, 3 direct observations, and semi-structured interviews with 18 purposively selected health professionals; content analysis	Hospital response during COVID-19	The system's capacity to maintain essential functions and absorb, adapt, and transform in response to unprecedented or unexpected change	Triangulation of documentary review, direct observation, and semi-structured interviews; content analysis organized into context, effects, strategies, and impacts, with strategies classified as absorption, adaptation, or transformation.
De La Garza 2022	France; La Pitié Salpêtrière Hospital in Paris	Qualitative case study; 37 explicitation interviews with 10 crisis-unit members, 17 clinical staff, and 10 administrative/support staff; remote observation of 35 crisis-unit meetings, documentary analysis	COVID-19 during the first national lockdown	The ability to anticipate disruptions so as to withstand them, adapt when necessary, and return to an acceptable state	Qualitative framework examining anticipation and in-situ adaptation across strategic, organizational, support-service, and frontline work; 13 crisis-management themes
**DeLuca 2025, 2026**	Italy; public and private hospitals nationwide	Quantitative study family using the same hospital-level survey base: 261 manager/director responses aggregated to 130 hospitals. The 2025 paper used structural equation modeling; the 2026 paper linked survey responses to Ministry of Health and AGENAS operational data and used moderated regression in 121 hospitals	During the COVID-19 pandemic	The capacity to withstand or absorb disruption, adapt and respond effectively, and maintain essential care and core organizational functions	Five-point survey measures of hospital resilience, digital technologies/ICTs, staff skills or digital skills, and internal and external information integration; the 2026 study added objective service-complexity and operational-efficiency indicators. Hospital resilience comprised six items in the 2025 model and seven indicators in the 2026 measurement model.
**Duvald 2025**	Denmark; five emergency hospitals (one university and four regional hospitals) in the Central Denmark Region	Qualitative comparative study; 13 semi-structured interviews involving 22 hospital, emergency-department, regional and prehospital managers/clinical leaders, purposively sampled	Before and during COVID-19	Maintaining positive adjustment under challenging conditions so that the organization emerges strengthened and more resourceful; examined through anticipation and coping during the first surge, with learning treated as an ongoing process	An open-ended interview guide based on multi-contingency organizational design was analysed using iterative open coding, after which anticipation, coping and ongoing learning were applied as a resilience framework; findings were validated against administrative documents
**Endaryono 2025**	Indonesia; 141 type C and D hospitals	Quantitative hospital-level survey using structural equation modelling, followed by interviews with seven hospital managers; 358 questionnaires collected, with 349 usable responses from 141 hospitals	Changing national health-insurance regulations and COVID-19	The ability to survive, adapt, recover, and potentially thrive during unexpected, disastrous, or turbulent events	Six-point questionnaire and structural equation model comprising six latent variables, 27 dimensions, and 103 observable variables. Strategic resilience was operationalized through perceived government regulation, organizational resources, strategic leadership, network capability, and resource reconfiguration, with hospital sustainability as the final outcome; seven manager interviews supported interpretation of the quantitative findings.
**Ezzati 2023**	Iran; health-system stakeholders at policy, health-care facility, and medical-university levels	Interpretive phenomenological qualitative study; semi-structured interviews with 59 participants (15 policymakers, 27 health-care facility managers/employees, and 17 medical-university faculty) selected through purposive and snowball sampling	During COVID-19	The ability and capacity to predict, prevent, prepare for, absorb, adapt to, and transform under shocks and stresses while continuing routine services during crisis management	43 perceived resilience challenges identified through semi-structured interviews and thematic analysis were organized into eight domains, from which a conceptual resilience model was developed
Fana 2021	South Africa; three public hospitals in a large rural and remote province	Comparative qualitative case study; 47 interviews with managers, shop stewards, and staff, plus 12 focus groups with 84 staff, using purposive and convenience sampling	Prolonged healthcare austerity & a provincial cost-containment process	The capacity to absorb, adapt, and transform under shock while retaining control over organizational structure and functions, supported by information use, anticipation of uncertainty, and management of interdependence	Interviews and focus groups examined hospital challenges, management responses, staff-management relations, and organizational functioning; strategies were interpreted as absorptive, adaptive, or transformative
**Fontana 2026**	Italy; 1,026 public and private accredited hospitals	Quantitative longitudinal hospital-month level panel study using linked administrative data for 1,026 hospitals from 2017–2022	Post- COVID-19 disruption	The capability to anticipate, respond effectively to, and recover rapidly from disruption while safeguarding continuity; empirically conceptualized as the restoration of elective hospital activity to pre-pandemic levels	Hospital-month Recovery Rate calculated as elective admissions in each month of 2021 or 2022 divided by the same hospital-month’s average admissions during 2017–2019; OLS models of log recovery included ownership, beds, staffing intensity, nearby-provider activity, provincial demographics and income, with year, month, and region fixed effects and hospital-clustered standard errors.
**Gautier 2023**	Brazil, Canada, France, and Japan; six hospitals	Qualitative international multiple-case study; 177 participants interviewed, including healthcare workers, senior and middle managers, clinical unit/department heads, supply unit heads, infection-prevention advisers, and project managers	Adaptation during COVID-19	Conceptualized through hospitals’ capacity to modify governance structures, including roles and responsibilities, and respond through shock absorption, adaptation, or transformation	Interviews were thematically analyzed using the HoSPiCOVID resilience framework and then re-analyzed using three European Observatory governance categories: a clear and timely response strategy, effective horizontal and vertical coordination, and clear and transparent communication.
**Ghahramani 2025**	Iran; seven major COVID-19 referral hospitals in Shiraz, Bushehr, and Tehran	Qualitative grounded-theory study using the Straussian approach; semi-structured, in-depth interviews with 12 hospital administrators, including managers, chiefs, and deputies; purposive followed by theoretical sampling	During COVID-19	A hospital’s ability to absorb disaster impacts without loss of function, maintain critical functions, and recover to its prior state or adapt to a new functional state	Semi-structured interviews examined hospital-management experiences, problems, and requirements during COVID-19 and were analyzed using constant comparison and open, axial, and selective coding.
**Gifford 2022**	Netherlands; five private nonprofit hospitals	Exploratory qualitative multiple-case study; 36 primary interviews with board members, managers, medical leaders, clinical staff, and supporting staff, preceded by 10 preliminary interviews; 548 pages of documents and 571 minutes of internal video	First and second COVID-19 waves	The dynamic capabilities that enable hospitals to sense environmental change, seize responses by reconfiguring resources, and transform organizational routines to cope with and recover from dynamic uncertainty	Grounded-theory-informed analysis of interviews, crisis-meeting records, strategic and policy documents, evaluations, and communications; adaptations were organized into decision-making, reorganization, human resources, material resources, and planning and then interpreted through sensing, seizing, and transforming capabilities.
**Gillberg 2023**	Sweden; one university hospital and one county hospital in western Sweden	Qualitative analysis embedded in a workforce survey; free-text responses from 4,097 managers and employees who answered the open-ended questions, representing 53% of 7,740 survey respondents and approximately 20% of 20,314 eligible employees	During the first COVID-19 peak	Positive adaptation to significant adversity through organizational systems that support anticipation and preparation before crisis, coping and solution implementation during crisis, and reflection, learning, and change afterward	Iterative qualitative analysis of responses to three open-ended survey questions, interpreted using Duchek’s capability-based anticipation, coping, and adaptation framework.
**Karim 2025**	United States; 2,243 rural nonfederal short-term general acute-care and Critical Access Hospitals	Quantitative cohort study with a pre-post design; 11,819 hospital-year observations from 2017–2022	During COVID-19	Not explicitly defined; financial resilience was conceptualized as the capacity to absorb pandemic-related financial shocks and maintain positive or comparatively stable profitability, reflected in pre-pandemic operating-margin categories and subsequent financial performance	Four profitability measures, patient-service operating margin, patient-service and hospital-operations margin, and total margins excluding and including PRF, were compared across 2017–2019, 2020, 2021, and 2022; hospitals were stratified by pre-pandemic patient-service operating margin as strained, vulnerable, or strong.
**Kim 2024**	United States; hospitals in Ohio, primarily one university-affiliated academic medical center comprising seven hospitals, with additional participants from other hospitals	Qualitative grounded-theory, multilevel study based on written essays from 30 hospital employees enrolled in a part-time MBA program at a Midwestern university; 21 worked at one university-affiliated academic medical center and nine at other hospitals	During COVID-19	Organizational capabilities to react to and recover from duress with minimal disruption to stability and functioning, encompassing anticipation, coping, recovery, and the possibility of thriving beyond the pre-crisis state	No formal resilience measure; written accounts were analyzed through open, axial, and selective grounded-theory coding, producing 25 first-order concepts, eight second-order themes, and four aggregate dimensions: resilience manifestation, leadership resilience and operational resilience.
Kjekshus 2023	Norway; Oslo University Hospital	Qualitative single-case study; 19 in-depth interviews and two focus groups involving staff across four hospital clinics and central HR, supplemented by internal audits, hospital documents, and public reports	Across COVID-19 waves	The capacity to cope with unanticipated dangers after they become manifest and to learn and bounce back, enabled by plastic, flexible structures and cross-boundary decision networks	Thematic and sensemaking analysis of interviews and documents contrasted a planned emergency logic based on anticipation, formal plans, and hierarchy with an ad hoc resilience logic based on shared understanding, improvisation, flexible authority, and rapid action.
Kyambade 2026	Uganda; two Regional Referral Hospitals in the Mbarara region	Quantitative cross-sectional survey using purposive sampling; 209 permanently employed nurses with at least two years of facility experience	Routine healthcare delivery.	The ability to anticipate, prepare for, respond to, and recover from disruptive events while sustaining operations and objectives; encompassing operational continuity, strategic adaptation, and employee psychological resilience	Organizational Resilience (Hartmann et al., 2020) and Resilient Leadership scales (Avey et al, 2009), and the World Health Organization Five Well-Being Index; relationships were analyzed using partial least squares structural equation modeling.
**Labarda 2017**	Philippines; one 275-bed public and one 125-bed private tertiary teaching hospital in Tacloban City	Exploratory retrospective mixed-methods case study; self-report preparedness assessment for both hospitals plus semi-structured interviews with five response leaders from each hospital	After Super Typhoon Haiyan (2013)	A dynamic, emergent process that reduces vulnerability and increases capacity to cope with unforeseen disturbance, sustain emergency services, and recover to the prior state or transform to a better state	Hospital Resilience Index (Zhong et al., 2015) based on 102 binary indicators across eight domains, hospital safety, disaster leadership and cooperation, disaster plan, emergency stockpiles and supply management, emergency staff, critical-care capability, training and drills, and recovery and reconstruction, plus an 11-item interview guide.
**Mohtady Ali 2023**	Australia; two public hospitals in Brisbane and the Gold Coast, Southeast Queensland	Qualitative case study using in-depth semi-structured interviews with 12 senior hospital directors, managers, and consultants who had operational leadership experience during disasters	Disasters and climate-related events	The ability to keep essential services operating with negligible disruption while responding to and recovering from concurrent disasters, with potential to adapt and transform beyond the prior performance level	No formal resilience measure; framework-informed thematic analysis of leadership experiences using the prevention–preparedness–response–recovery cycle and resilience capacities.
**Moitinho de Almeida 2021**	Nepal; Tribhuvan University Teaching Hospital (TUTH), a tertiary referral hospital in Kathmandu	Qualitative single-hospital study; 18 semi-structured interviews with clinical, managerial, technical, and support staff	After the 2015 Nepal earthquake	The ability to overcome disaster through inherent capacity and adaptive flexibility, meet emergency demand, and retain essential functions; framed through redundancy and resourcefulness as means and robustness and rapidity as outcomes	Combined inductive thematic analysis of hospital burden and individual staff resilience with deductive coding using the four system-resilience dimensions: redundancy, resourcefulness, robustness, and rapidity.
**Moradi 2022**	Iran; Shiraz University of Medical Sciences and affiliated hospitals designated as COVID-19 centers	Qualitative content-analysis study using semi-structured interviews with 17 purposively and conveniently selected university and hospital personnel, including managers, nurses, physicians, specialists, and quality, infection-control, and patient-safety staff	During COVID-19.	Conceptualized as maintaining quality care and continuing hospital services during an epidemic despite rapidly rising demand and operational disruption	No formal resilience or service-quality measure; semi-structured interviews were analyzed using Graneheim and Lundman’s qualitative content-analysis method in MAXQDA 2020, producing five themes and 31 subthemes.
**Mukherjee 2025**	India; 193 public and private hospitals	Quantitative cross-sectional survey; 193 usable responses from hospital managers, medical superintendents, information technology (IT), and other mid- to senior-level operational leaders	Disruptions in inpatient care	Operational resilience was conceptualized as mitigating disruptions and adapting through dynamic reconfiguration of human, technical, and procedural resources	Survey constructs measured artificial intelligence (AI) integration within knowledge-management systems (KMS), six-item disruption impact, resource reconfiguration, trust in AI, and technical complexity; two simultaneous equations were estimated using Three-Stage Least Squares (3SLS).
**Neupane 2024**	United States; 620 hospitals in the PINC AI Healthcare Database	Retrospective cohort study of 223,380 adult inpatients with COVID-19 across 620 hospitals: 208 extracorporeal membrane oxygenation-capable hospitals, 216 hospitals with multiple intensive care unit (ICU) types, 36 hospitals with at least 200 beds and one ICU, and 160 hospitals with fewer than 200 beds and one ICU	During Delta-wave COVID-19 surges	Not explicitly defined as a general organizational construct; resilience was represented by the extent to which hospital care quality and patient survival were maintained as severity-weighted COVID-19 caseload increased relative to bed capacity	Previously validated hospital-month surge index measuring severity-weighted COVID-19 inpatient caseload relative to prepandemic bed capacity; hierarchical generalized linear models estimated adjusted in-hospital death or hospice-discharge probability, with effect modification tested across four hospital infrastructure types.
Qassim 2024	Saudi Arabia; public and private hospitals across the country.	Quantitative cross-sectional survey; stratified random sample of 127 hospital administrative staff	Routine hospital operations	The ability to anticipate, prepare for, respond to, and adapt to gradual changes and sudden disruption so the hospital can survive, thrive, maintain operations, and continue quality care	Structured questionnaire comprising 13 occupational-health-and- safety items, nine organizational-resilience items (Kantur & Say, 2015).
**Qiao 2023**	United States; eight South Carolina organizations providing HIV services, comprising six AIDS Service Organizations, one academic medical center, and one state public-health agency	Qualitative study using 10 in-depth interviews with 11 organizational leaders from eight HIV service organizations [*one interview included two participants*]	During COVID-19	The ability to maintain needed health services amid rapidly changing circumstances	No formal resilience measure; semi-structured leader interviews were analyzed thematically using a codebook informed by the interview guide, literature, early interviews, and subsequently emergent themes.
**Ravaghi 2023**	Eastern Mediterranean Region; hospitals represented across 18 of 22 countries, including high-, middle-, and low-income, conflict-affected, and resource-constrained settings	Multi-method regional study triangulating a scoping review, 64 semi-structured key-informant interviews selected through purposive maximum-diversity sampling, and 252 survey responses across two phases	COVID-19	Hospital resilience comprises six interdependent components (space, stuff, staff, systems, strategies, and services) and absorptive, adaptive, transformative, and learning capacities that enable essential functions to continue and the hospital to recover efficiently to its prior or an improved state	Thematic content analysis of interviews and open-ended survey responses with descriptive survey analysis. Hospital resilience was assessed using author-developed Likert items based on the six-component/four-capacity framework, WHO hazard categories, and disaster-risk-management stages
**Righi 2024**	Brazil; public, public-private, and private hospitals, predominantly in the South and Southeast regions	Mixed-methods descriptive exploratory study using a snowball convenience survey of 111 frontline healthcare workers and five follow-up questerview interviews with one physician, one physiotherapist, two nurses, and one nursing technician	During the first COVID-19 waves.	The ability to adjust functioning before, during, or after changes and disturbances so required performance can be sustained	Adapted Resilience Analysis Grid (RAG) questionnaire assessing five resources for action, information and communication; team, equipment, and tools; standard operating procedure (SOP); training; and built environment, across anticipating, monitoring, responding, and learning using a five-point agreement scale. Descriptive survey analysis was supplemented by framework-guided thematic analysis of the questerviews.
Rosenbäck 2025	Sweden; two public hospitals in different regions: one medium-sized emergency hospital in an urban region and one large university hospital	Comparative qualitative two-case study; semi-structured video interviews with 41 hospital and regional executives, crisis-management personnel, clinical and departmental managers, and support-function leaders ,27 in Case A and 14 in Case B, supplemented by secondary sources	During COVID-19	Not explicitly defined; treated as organizational learning and adaptive response during crisis, with resilience shaped by power relations, sense giving, and the interaction and integration of formal and informal leadership	Resilience was interpreted qualitatively through deductive thematic analysis of interviews and secondary sources using organizational-learning and learning-cycle, power-game, sense giving, and power and leadership-dynamics concepts, presented through three critical narrative episode
**Sari 2024**	Indonesia; hospital in Makassar, South Sulawesi	Qualitative study; semi-structured interviews with 20 purposively selected hospital policymakers, managers, and hospital-resilience experts with at least 10 years of working experience	During technological, social, economic, political, and market disruptions	The overarching goal of continuing to function as effectively as possible under pressure; conceptualized as a function of vulnerability and adaptability, encompassing shocks or stresses, capacity to manage disturbance, and outcomes ranging from transformation or improved recovery to collapse	Hospital-resilience was interpreted qualitatively through semi-structured interviews, deductive and emergent thematic coding and mapping to an adapted WHO resilience framework
**Schlinkert 2024**	Netherlands; nine hospitals - two academic, four top-clinical, and three general hospitals	Mixed-methods interview study combining oral resilience ratings with qualitative data; semi-structured video interviews with nine hospital board members, one from each hospital	The first 14 months of COVID-19	Organizational capacity for handling everyday tasks and performing during crisis situations, including learning from crises, identifying risks and vulnerabilities, and preparing for future emergencies	Dutch forward–backward translation of the 13-item Benchmark Resilience Tool short form developed by Whitman, based on Seville and colleagues’ organizational-resilience indicators; supplemented by qualitative probing and thematic content analysis
**Shi 2024**	China; hospital in Wuhan	Qualitative interview study; purposive and snowball sample of 26 staff, including nurses, physicians, administrators, pharmacists and dietitians, radiology and laboratory technicians, an ultrasonography worker, and a traditional Chinese medicine practitioner	During the initial COVID-19 outbreak in Wuhan	Conceptualized as a dynamic adaptive process in which hospitals mobilize protective factors in response to risks and disruption, producing states of imbalance, obstruction, recovery, or activation, while theoretically embedded within broader community and healthcare-system levels	Semi-structured interviews were guided by the WHO infectious-disease Action Framework, with protective and risk factors identified through manual deductive and inductive template analysis.
**Soleimani 2025, 2026**	Iran; hospital-network experts and hospital professionals in Isfahan	Sequential mixed-methods study family, literature review and semi-structured interviews with 13 experts or senior healthcare professionals, followed by a cross-sectional survey of 169 hospital professionals	Overall disruptive events, including pandemics	The capacity to anticipate or forecast, absorb, adapt to, transform in response to, withstand, and recover from disruptions while maintaining critical operations or restoring pre-crisis functioning	Thematic/content analysis identified eight dimensions. Organizational resilience was measured with four items covering predictive or forecasting, absorptive, adaptive, and transformative capacities. Related structural and mediation models were analyzed using partial least squares structural equation modeling (PLS-SEM) in SmartPLS
**Son 2020**	United States; one large regional hospital in southeastern Texas	Qualitative case study; semi-structured interviews with six incident-command participants in executive, operations, health and safety, compliance, environmental health and safety, and facilities-management roles	During Hurricane Harvey	The ability of a social and physical system to prepare for, respond to, and recover from disaster effects in a timely and efficient manner	Thematic qualitative analysis across six domains: organizational structure and roles, communication and situational awareness, operating plans and protocols, human and physical resources, lessons from previous incidents, and leadership and high-level decision-making
**Sutton 2025**	England; three large city-based National Health Service (NHS) teaching hospital trusts with across seven hospital sites	Qualitative multi-site study; observations of 29 operations-management meetings plus 17 semi-structured interviews with divisional leads, managers, and meeting chairs	Under chronically high operational pressure	Conceptualized as sustaining everyday organizational performance and service delivery through adaptations and adjustments to fluctuating demand, capacity, and other expected or unexpected pressures	Structured meeting observations and interviews were analyzed iteratively using a modified grounded-theory approach, with resilience interpreted through sense-making and interpretation, risk work, and morale maintenance
**Tongtong 2024**	China; primary/community, secondary, and tertiary public hospitals recruited through the China Public Hospital Management Conference	Quantitative cross-sectional online survey; purposive sample of 197 public-hospital managers and staff	COVID-19	The ability to resist, absorb, adapt to, and recover from adverse events while maintaining essential services and achieving rapid recovery and development	Literature-based seven-point Likert questionnaire measuring supply-chain strategy formulation, leadership, digital-technology capability, risk-control capability, and hospital resilience; Relationships were analyzed using partial least squares structural equation modeling (PLS-SEM) in SmartPLS 4.1
**Tongtong 2025**	China; primary, secondary, and tertiary public hospitals in seven eastern, central, and western provinces	Quantitative cross-sectional survey; purposive non-probability sample of 249 valid respondents distributed through a hospital-management conference work group	Following COVID-19	An organization's ability to resist, adapt, and recover during emergencies; public-hospital resilience was represented through robustness, adaptability, and flexibility	Hospital resilience was measured with six items representing robustness, adaptability, and flexibility. Relationships were analyzed using partial least squares structural equation modeling (PLS-SEM) in SmartPLS 4
**Tortorella 2021**	Multinational; 109 Healthcare 4.0 and resilient-healthcare experts and practitioners from emerging and developed economies	Exploratory quantitative expert survey; 109 complete responses	Routine	The ability to adjust functioning before, during, or after changes and disturbances to sustain required performance, supported by the abilities to monitor, anticipate, respond, and learn	Online survey assessing the perceived impact of 10 Healthcare 4.0 technologies on monitoring, anticipating, responding, and learning using a five-point scale from very weak to very strong impact, with a no-impact option; hierarchical cluster analysis and tests of cluster-mean differences.
**Tortorella 2022**	Brazil; one public teaching hospital with 257 inpatient and 45 intensive care unit (ICU) beds and one private hospital with 119 inpatient and 29 ICU beds	Qualitative multiple-case study; semi-structured interviews with 10 senior leaders (five from clinical and five from non-clinical departments)	During COVID-19	The ability of a healthcare system to adjust its functioning before, during, or after changes and disturbances so that required performance is sustained under expected and unexpected conditions; examined through the abilities to monitor, anticipate, respond, and learn	Semi-structured interview data were analyzed through qualitative content analysis, with Healthcare 4.0 applications categorized as supply chain, patient diagnosis, patient treatment, or patient follow-up and mapped to the four resilience abilities
**Yun 2025**	South Korea; 21 private long-term care hospitals, most with fewer than 300 beds	Sequential mixed-methods study; online survey of 21 infection-control professionals representing 21 hospitals, followed by 11 semi-structured interviews with frontline leaders and middle managers from selected hospitals	During COVID-19	A system’s capacity to promptly and effectively withstand, absorb, adapt to, and recover from hazards while maintaining critical functions	Author-developed and expert-reviewed survey based on national business-continuity guidance, the WHO Hospital Emergency Response Checklist, and national medical-institution guidelines; Descriptive statistics were integrated with thematic analysis of interviews

**Table 2A.** Qualitative evidence on organizational resilience factors

Study	Organizational factors and capabilities identified	Role	Key findings
**Cannedy 2022**	F: workforce resources; digital technologies; organizational structures; policy support. C: absorb disruption; adapt.	Facilitators and resilience-supporting actions	Clinical Resource Hubs rapidly redeployed virtual contingency providers to maintain outpatient, inpatient, and triage services, including primary care, mental-health and specialty coverage, call-center support, and care outside the Veterans Health Administration. Hub staff also trained providers in virtual care and supported employees through coaching, counseling, and health screening. Embedded staffing flexibility, existing telehealth expertise, and increased acceptance of virtual care facilitated the response, which the authors interpreted as supporting adaptability, self-regulation, integration, and staff well-being.
**Cuno-Chunga 2025**	F: Organizational culture; communication; leadership support C: Adapt and reconfigure; Learn and transform	Facilitators	Participants described proactive and collaborative adaptation, emotional self-regulation, respectful communication, teamwork, and practical innovation as sustaining hospital functioning. Communication emerged as the most prominent theme, while supportive leadership and an institutional culture open to change facilitated technology adoption, workflow adaptation, and continuity of service.
de Almeida Marques 2026	F: Leadership and decision authority; organizational structures and coordination arrangements; communication C: Adapt and reconfigure; Learn and transform	Barriers and compensatory facilitators	Central–local misalignment, contradictory directives, ambiguous authority, and perceived political pressure delayed decisions and weakened coordination, trust, and organizational learning. Informal departmental leadership and peer networks enabled rapid local adaptation when formal channels were unresponsive. At CMIN, some bottom-up practices were incorporated into formal contingency plans, improving coordination in later phases.
**de Araujo Oliveira 2023**	F: leadership; workforce resources; well-being; communication; preparedness arrangements; material resources; financial resources; infrastructural resources; external networks. C: prepare; absorb disruption; adapt; reconfigure; learn; transform.	Facilitators and barriers	Four mostly favorable resilience configurations were identified. Absorption and transformation occurred early, while adaptation became more prominent as cases increased. The hospital rapidly expanded and reorganized services through infrastructure changes, protocols, staffing measures, training, testing, and community support. Material shortages, workforce disruption, communication difficulties, clinical uncertainty, and reduced non-COVID-19 services exposed continuing weaknesses.
De La Garza 2022	F: leadership; workforce resources; well-being; communication; preparedness arrangements; organizational structures; coordination arrangements; material resources. C: anticipate; prepare; adapt; reconfigure; learn; transform.	Facilitators and barriers	Resilience arose from continuous trade-offs between planned anticipation and frontline adaptation. Linking crisis-unit decisions with operational realities enabled rapid changes to beds, staffing, and protocols. Collective expertise, coordination, and support facilitated adaptation, while shortages, clinical uncertainty, workload, and emotional strain constrained it.
**Duvald 2025**	F: governance; decision authority; information systems; communication; preparedness arrangements; organizational structures; coordination arrangements; workforce resources; external networks. C: anticipate; prepare; adapt; reconfigure; learn.	Mixed	Four of five hospitals activated general catastrophe plans, but these were not designed for a prolonged pandemic. Hospitals therefore repeatedly modified governance and clinical operations through task-force and Pandora structures, different patient-care configurations, expanded information systems and regional capacity coordination. Resilience was linked to the ability to recognize organizational misfits, delegate decisions appropriately, learn and make real-time adjustments.
**Ezzati 2023**	F: leadership; governance; workforce resources; organizational culture; information systems; communication; preparedness arrangements; organizational structures; coordination arrangements; material resources; financial resources; external networks; community relationships.	Barrier	Participants described fragmented leadership and coordination, inadequate national and operational planning, limited preparedness and organizational learning, workforce and community-engagement weaknesses, insufficient resources and information systems, and poor process monitoring. Suggested responses included integrated evidence- and community-based management, clearer command structures, workforce development, stronger information and resource capacity, and process redesign.
Fana 2021	F: leadership; communication; workforce resources; material resources; financial resources. C: absorb disruption; adapt; transform.	Facilitators and constraints	The hospital with participatory leadership, intensified communication, joint planning, labor-relations training, and new engagement forums developed workable local responses and functioned more effectively. In hospitals characterized by autocratic leadership and poor communication, reliance on absorptive strategies generated resistance, increased sick leave, mistrust, interdepartmental conflict and weaker organizational resilience.
**Gautier 2023**	F: governance; decision authority; organizational culture; communication; preparedness arrangements; coordination arrangements. C: anticipate; adapt; reconfigure.	Facilitators and barriers	Cross-site variation was attributed primarily to pre-existing managerial openness and routine preparedness planning and training. Multidisciplinary decision bodies, trusted coordination across levels, and rapid transparent communication were perceived to support timely responses, whereas top-down processes and unclear roles reduced staff inclusion and acceptance. Hospital-wide updates combined with targeted team channels were perceived to minimize information asymmetry, although information overload persisted in several hospitals. Where managerial openness was weaker, frontline criticism was more pronounced.
**Ghahramani 2025**	F: decision authority; workforce resources; communication; material resources; financial resources; infrastructural resources. C: adapt; reconfigure	Barriers and response strategies	Lack of hospital resilience emerged as the core management challenge. Financial, infrastructural, human-resource, communication, supply-chain, and decision-making weaknesses constrained hospitals’ responses. Managers used specialist-role redesign, compensation priorities, external and community resources, and locally innovative infrastructure and care solutions; however, inadequate documentation and information sharing were linked to failed innovations and persistent or recurring problems.
**Gifford 2022**	F: workforce resources; preparedness arrangements; material resources; external networks. C: anticipate; prepare; adapt; reconfigure; learn.	Proposed facilitators and constraints	All five hospitals reported insufficient preparedness and initially responded largely reactively, but rapidly centralized decisions, expanded COVID-19 capacity, separated patient flows, redeployed staff, and adapted resource sourcing. Changes between the first and second waves demonstrated organizational learning, although hospitals struggled to retain gains in collaboration and rapid decision-making. The authors interpreted resilience as requiring a balance between efficiency and flexibility through stronger sensing, seizing, and transforming capabilities.
**Gillberg 2023**	F: organizational culture; communication. C: absorb disruption; adapt; reconfigure; learn.	Facilitators, barriers, and organizational tensions	Four interrelated processual practices constructed resilience capability: problem-solving orientation, cooperation and trust, organizational learning, and information and communication. The crisis enabled rapid frontline action, prioritization of patient care, cross-boundary cooperation, and knowledge acquisition, while unclear, contradictory, excessive, or insufficient information impeded resilience. Resilience was also characterized by tensions between flexibility and structure, hierarchical communication and peer dialogue, and acute priorities and continuing core operations; sustainable practices required interaction across individual, team, and organizational levels.
**Kim 2024**	F: leadership; communication. C: adapt; reconfigure.	Multilevel precursors and resilience manifestations	Resilience was interpreted as emerging through coordinated activity across organizational levels. Top management maintained communication and conveyed strategic direction, while middle managers attended to individual needs, supported emotional stability, and developed group cohesion. Early improvised changes in jobs, staffing, training, and physical arrangements were followed by more deliberate compensation, leave, job-security, and policy changes. These leadership, HRM, and individual-level attributes were incorporated into a proposed process model preceding reported recovery in revenue and productivity.
Kjekshus 2023	F: governance; decision authority; communication; preparedness arrangements; organizational structures; coordination arrangements; financial resources. C: anticipate; prepare; adapt; reconfigure.	Ad hoc flexibility: facilitating; rigid planned logic: constraining.	Ad hoc resilience logic dominated the initial phase, supporting rapid decisions, staff redeployment, infection-control reconfiguration, and on-site coordination despite inadequate preparation for staff-to-staff transmission. As the crisis persisted, shared understanding weakened and staff increasingly demanded predictable plans, reducing support for improvisation. Ad hoc organizing was considered effective but exhausting, indicating the need to balance planning with flexible sensemaking.
**Labarda 2017**	F: leadership; governance; workforce resources; preparedness arrangements; organizational structures; coordination arrangements; material resources; financial resources; infrastructural resources; external networks; community relationships. C: anticipate; prepare; absorb disruption; adapt; reconfigure; restore; recover.	Facilitators and constraints	Both severely damaged hospitals initially delivered emergency care, demonstrating that resilience was a dynamic process rather than a static preparedness score. Long-term recovery diverged: the public hospital restored normal or near-normal services within weeks, supported by staff commitment, internal capacity, and sustained government and partner assistance. The private hospital initially continued emergency care but exhausted supplies, financing, and human resources and later ceased inpatient operations amid limited sustained support and organizational restructuring. Preparedness needed to be embedded in everyday organizational practice.
**Mohtady Ali 2023**	F: leadership; governance; decision authority; communication; preparedness arrangements. C: anticipate; prepare; absorb disruption; adapt; learn.	Proposed resilience-enabling competencies	The seven competencies were interpreted as enabling hospital disaster resilience through decisive and accountable leadership, anticipation and navigation of evolving risks, adaptable planning, timely communication, collaboration, and learning from internal and external experience. The proposed Anticipate–Assess, Prevent–Prepare, Respond–Recover, Reflect–Regenerate, Apply–Assess model connects these competencies to actions before, during, and after disasters.
**Moitinho de Almeida 2021**	F: leadership; governance; decision authority; communication; preparedness arrangements. C: anticipate; prepare; absorb disruption; adapt; learn.	Facilitators and constraints	The hospital avoided complete functional loss and expanded emergency capacity by suspending elective care, discharging stable patients, and reorganizing staff and services, although routine activities and quality of care were affected. Surgery began within two hours, and the major victim burden was addressed over approximately two to three weeks. Emergent adaptations were central to redundancy and resourcefulness, while staff safety, meaningfulness, and belonging supported a sustainable response.
**Moradi 2022**	F: workforce resources; well-being; material resources; infrastructural resources; external networks. C: adapt; reconfigure.	Perceived facilitators and successful practices	University support coordinated referral designation, private-hospital capacity, outpatient care, volunteers, donors, and interorganizational collaboration. Hospitals expanded bed and specialized-care capacity, strengthened infection control, triage, screening, remote care, equipment and oxygen supply, redeployed and trained staff, and provided accommodation and psychological support. Participants regarded these practices as helping maintain service quality during COVID-19 and as lessons for future epidemic preparedness.
**Qiao 2023**	F: communication; preparedness arrangements; leadership; well-being; material resources; financial resources; digital technologies; external networks. C: prepare; adapt; reconfigure.	Facilitators when available and effective; barriers when absent, inadequate, or inflexible	Coordinated leadership responses, accurate information, and protocols that could be revised as needs emerged helped organizations adapt HIV services during COVID-19. Staff psychological support, access to PPE, interorganizational sharing of scarce supplies, and flexible funding supported continued operations. Telehealth enabled continuity in some organizations, while rigid line-item funding, supply shortages, inadequate infrastructure, and unsuccessful telehealth implementation constrained adaptation.
**Ravaghi 2023**	F: leadership; decision authority; workforce resources; well-being; communication; preparedness arrangements; coordination arrangements; financial resources; material resources; external networks; community relationships. C: prepare; absorb disruption; adapt; reconfigure; recover; learn; transform.	Facilitators and constraints	Hospitals supported COVID-19 recovery through community education, risk reduction, and continuity of essential services. Respondents emphasized workforce mobility, competence and protection, reserve and diversified financing, adaptive leadership, learning systems, and coordinated technical and logistical support from ministries of health and the WHO. Hospital resilience was viewed as dependent on integration with communities and the wider health system rather than isolated hospital action.
**Righi 2024**	F: workforce resources; communication; preparedness arrangements; material resources; infrastructural resources. C: anticipate; prepare; adapt; reconfigure; learn.	Interdependent facilitators when available and adaptable; constraints when insufficient	Frontline workers described learning unfamiliar roles, revising procedures, expanding and adapting clinical spaces, and relying on rapid communication and multidisciplinary teamwork. Training was particularly important for learning, while reliable and current information supported rapid response and monitoring. The five resource categories operated interdependently: changes in spaces, staffing, equipment, procedures, and training required corresponding changes in communication and coordination.
Rosenbäck 2025	F: leadership; decision authority; communication; preparedness arrangements; organizational structures; coordination arrangements. C: absorb disruption; adapt; reconfigure; learn.	Facilitators and barriers	Participants described misaligned top-down directives, unclear authority, missing information, and poor documentation as disrupting coordination and organizational learning. Hospital and department managers bypassed or supplemented regional structures to address immediate operational needs, but these informal arrangements generated tension with formal management. Later formalization of local crisis management and integration of the department-manager group improved role clarity, documentation, communication, and decision-making. The authors concluded that resilience requires combining formal authority with flexible, frontline-informed action and integrating effective informal arrangements into formal crisis-management structures
**Sari 2024**	F: leadership; workforce resources; organizational culture; external networks.	External disruptions and internal barriers	Participants described hospital disruption as extending beyond disasters to policy instability, management-paradigm shifts, changing markets and consumer behavior, technological advances, politicization, disasters, and economic conditions or conflict. They identified organizational culture, weak cross-sector cooperation, traditional management, inadequate managerial, leadership, and human-resource capabilities, limited business orientation, and resistance to change as barriers to hospitals becoming resilient. The authors interpreted these internal conditions as potential constraints on adaptive capacity and proposed that the identified shocks and barriers inform subsequent development of hospital-resilience capabilities and indicators.
**Schlinkert 2024**	F: leadership; decision authority; workforce resources; organizational culture; communication; preparedness arrangements; material resources; financial resources; external networks. C: anticipate; prepare; adapt; reconfigure; learn.	Facilitators and barriers	Board members perceived their hospitals as well prepared for short, acute crises through clear leadership, continuous communication, staff autonomy, rapid decision-making, and strong networks. Reflection and revision of processes between infection waves were viewed as supporting smoother anticipation and response. As the pandemic continued, cohesion and proactiveness declined, staffing and resource constraints became more prominent, and hospitals struggled to operate crisis care and regular services in parallel. The authors emphasized maintaining trust and bounded staff autonomy while monitoring external threats and translating lessons into future preparedness.
**Shi 2024**	F: decision authority; workforce resources; communication; digital technologies; preparedness arrangements; coordination arrangements; material resources; infrastructural resources. C: adapt; reconfigure; learn.	Protective factors and risks	Hospital response was better supported when temporary teams had clear roles and effective coordination, resources were adjusted dynamically, monitoring was timely but proportionate, communication was synchronized and transparent, and clinical and public-health functions were supported by training, prior infectious-disease experience, telemedicine, adaptable spaces, protective equipment, and infection-control systems. Unfamiliar team members could create command difficulties, repetitive monitoring increased workload, and isolation and protective equipment impeded communication.
**Soleimani 2025, 2026**	F: information systems; communication; digital technologies; preparedness arrangements. C: anticipate; prepare; absorb disruption; adapt; reconfigure; recover; transform.	Proposed facilitators	Eight interconnected dimensions comprising CRM resources, organizational capabilities, risk and crisis management, and hospital resilience. Customer knowledge and CRM technology were positioned as resources supporting dynamic, innovation, and flexibility capabilities; risk and crisis management connected these capabilities to predictive, absorptive, adaptive, and transformative resilience.
**Son 2020**	F: leadership; decision authority; workforce resources; well-being; information systems; communication; preparedness arrangements; organizational structures; coordination arrangements; material resources; infrastructural resources; external networks. C: prepare; absorb disruption; adapt; reconfigure; learn.	Opportunities and challenges	The hospital established two incident command centers, shifted operations toward outpatient care, maintained critical patient reporting, adapted staffing and space, mobilized backup equipment and supplies, and applied lessons from previous storms through floodgates, relocated equipment, inspections, and drills. The two-command-center structure also increased coordination demands. Participants described indirect inter-hospital communication, incompatible emergency and clinical information systems, difficulty applying formal planning and reimbursement procedures, staff fatigue and anxiety, hazards associated with improvised resources, delayed emergency-plan activation, and limited regional learning and leadership as continuing challenges.
**Sutton 2025**	F: workforce resources; information systems; coordination arrangements. C: anticipate; adapt; reconfigure.	Facilitators	Operations meetings brought clinical and operational staff together up to four times daily to combine centralized data with local intelligence, develop a shared understanding of hospital pressures, identify and prioritize risks, and allocate actions to maintain patient flow. Sense-making, interpretation, and risk work supported organizational functioning, while recognition, empathy, and mutual support helped sustain morale and individual staff resilience under persistent pressure.
**Tortorella 2022**	F: digital technologies. C: anticipate; adapt; learn.	Facilitators, with contributions varying by resilience ability	Supply-chain and diagnostic applications strongly supported monitoring. Supply-chain, diagnostic, and treatment applications supported anticipation, while supply-chain and treatment applications only partially supported response. Diagnostic, treatment, and follow-up applications supported learning. Patient-follow-up applications did not support anticipation or response. The private hospital reported broader digital adoption and stronger support for response, reflecting differences in ownership, investment capacity, and implementation maturity.
**Yun 2025**	F: leadership; organizational culture; workforce resources; communication; preparedness arrangements; financial resources; material resources; infrastructural resources; external networks. C: prepare; adapt; reconfigure.	Facilitators and barriers	Six themes explained how hospitals maintained operations. Leadership, staff cooperation, informal communication networks, service prioritization, workforce adaptation, infection-control measures, and resource management supported continuity. Inadequate operational planning, staffing shortages, fragmented external communication, transfer delays, financial constraints, and unstable supply arrangements impeded it.

**Note:** F = organizational factor; C = resilience capability. The factor and capability terms listed for each study reflect the specific component(s) supported by that study and map to the six higher-order factor domains and five higher-order capability domains used in the synthesis.

**Table 2B.** Quantitative evidence on organizational resilience factors

Study	Organizational factors and capabilities examined	Direction/association	Key findings
Afsar 2026	F: governance; coordination arrangements. C: adapt.	Positive for most paths; responsibility–planning nonsignificant; private > Ministry of Health > university hospitals	Self-organization showed the strongest reported association with adaptive capacity (β = 0.654, p < 0.001). In the aggregated three-category comparison, employee-reported governance and resilience scores were highest in private hospitals, followed by Ministry of Health and university hospitals.
Al-Ayed 2019	F: workforce resources	Positive and statistically significant across all resilience dimensions	Overall SHRM was positively associated with cognitive (β=0.31), behavioral (β=0.40), and contextual organizational resilience (β=0.38), all p<0.05.
**Atkinson 2024**	F: communication; organizational structures. C: anticipate; adapt.	Formality and informality → higher resilience; informality stronger	Formality was positively associated with resilience at the 25th, 50th, and 75th quantiles (b = 0.290, 0.253, and 0.182), while informality showed consistently larger associations (b = 0.642, 0.676, and 0.586; all p < .001). Relational interactions (b = 0.278, p < .001), transactional interactions (b = 0.264, p < .001), and ad hoc collaboration (b = 0.144, p < .01) were positively associated with resilience.
**Bosque-Mercader 2025**	F: workforce resources; infrastructural resources.	Most factors → no systematic association	English NHS acute trusts recovered an average of 83.2% of total elective activity in 2021 relative to 2019. Most supply and demand characteristics were not systematically associated with recovery. Compared with London, total elective recovery was 22.0% lower in North East and Yorkshire and 19.0% lower in North West; lower recovery was also observed in East of England, the Midlands, and South West. Regional differences were particularly evident for orthopedic and vascular surgical activity
**DeLuca 2025, 2026**	F: workforce resources; communication; digital technologies; external networks.	Mixed and context-dependent	2025: digital technologies (β=0.273, p<0.001) and external information integration (β=0.366, p<0.001) were positively associated with hospital resilience, whereas staff skills and internal information integration had no direct effects. External information integration partially mediated the digital-technology relationship and fully mediated the staff-skills relationship. The 2026 study added that operational-efficiency indicators moderated the relationships of ICTs and digital skills with resilience, while case mix strengthened the relationship between internal information integration and resilience; external-information-integration effects were not moderated.
**Endaryono 2025**	F: leadership; financial resources; organizational culture. C: reconfigure.	Mixed	Organizational resources (β=0.51) and perceived regulation (β=0.20) were positively associated with strategic leadership. Strategic leadership was positively associated with resource reconfiguration (β=0.60) and hospital sustainability (β=0.30), while resource reconfiguration was positively associated with sustainability (β=0.53). Strategic leadership was negatively associated with network capability (β=−0.79), and network capability was negatively associated with resource reconfiguration (β=−0.39).
**Fontana 2026**	F: workforce resources; infrastructural resources.	Mixed	Public hospitals had lower recovery than accredited private hospitals in both descriptive and adjusted analyses: average Recovery Rates were 0.80 versus 0.94 in 2021 and 0.91 versus 0.98 in 2022, while the pooled adjusted public-hospital coefficient was −0.213 (p < .01). Higher staffing intensity was positively associated with recovery. Nearby private-provider activity was negatively associated with recovery at 50- and 70-km radii but not at 30 km. Hospitals serving older populations (age 65+) was associated with weaker recovery mainly in 2022.
**Karim 2025**	F: financial resources; policy support.	PRF: positive; 2020 pandemic year: negative.	Eighty-five percent of rural hospitals were financially strained or vulnerable before COVID-19, and patient-service operating margins declined across all three groups in 2020. Among strained hospitals, mean total margin including PRF reached 8.39% in 2021 but declined to 0.76% in 2022; vulnerable hospitals showed a similar temporary improvement, while strong hospitals maintained positive total margins without PRF. PRF-inclusive margins indicate short-term financial support rather than resolution of underlying financial fragility.
Kyambade 2026	F: leadership; well-being.	Positive direct associations and a significant indirect association through nurses’ well-being	Resilient leadership was positively associated with organizational resilience (β = 0.452, p < 0.001) and nurses’ well-being (β = 0.658, p < 0.001), while nurses’ well-being was positively associated with organizational resilience (β = 0.829, p < 0.001).
**Labarda 2017**	F: leadership; workforce resources; preparedness arrangements; material resources	Higher HRI → stronger recovery	The public hospital’s HRI was 0.72 versus 0.28 for the private hospital and was higher in all eight domains. The public hospital maintained service delivery and restored normal or near-normal operations within weeks, whereas the private hospital initially provided emergency care but exhausted supplies, financing, and human resources and later ceased inpatient operations [*this was a descriptive comparison of two self-assessed hospitals, not a statistically tested association*].
**Mukherjee 2025**	F: digital technologies. C: reconfigure.	AI-KMS integration → lower disruption and higher reconfiguration; complexity → weaker mitigation; trust → stronger reconfiguration.	AI-KMS integration was associated with lower disruption impact (β = −0.460, p < 0.10) and greater resource reconfiguration (β = 0.614, p < 0.10). Technical complexity weakened the negative AI–disruption association (interaction β = 0.067, p < 0.01), while trust strengthened the AI–reconfiguration association (interaction β = 0.125, p < 0.01).
**Neupane 2024**	F: infrastructural resources.	Higher caseload strain → higher death or hospice-discharge probability	Each one-unit increase in the log-transformed surge index was associated with a 5.51-percentage-point increase in the adjusted probability of in-hospital death or hospice discharge (95% confidence interval, 4.53–6.50). An estimated 7,375 events were potentially attributable to caseload surge, representing 21.5% of the primary-outcome events. The surge–outcome association did not significantly differ across the four hospital infrastructure types (interaction p = 0.32), providing no evidence that more advanced hospitals were less affected by caseload strain.
Qassim 2024	F: well-being.	OHS → higher SE, SR, and HOR; SE and SR → higher HOR; serial mediation positive.	OHS was positively associated with SR (β = 0.608, p < 0.001), SE (β = 0.568, p < 0.001), and HOR. SR (β = 0.232, p = 0.008) and SE (β = 0.591, p < 0.001) were also positively associated with HOR. The serial indirect effect through SE and SR was significant (β = 0.081, p = 0.016; 95% CI 0.019–0.143), while the direct effect remained significant, supporting partial mediation.
**Righi 2024**	F: workforce resources; communication; preparedness arrangements; material resources; infrastructural resources. C: anticipate; learn.	Positive; learning highest, anticipating lowest; information and communication highest	Mean perceived resilience was 4.15 on a five-point scale (standard deviation (SD) 0.91). Learning received the highest rating at 4.23 (SD 0.96), followed by responding at 4.08 (SD 1.02), monitoring at 3.85 (SD 1.07), and anticipating at 3.70 (SD 1.11). Information and communication was the highest-rated resource at 4.20. Combinations of resources were perceived as supporting adaptive performance.
**Schlinkert 2024**	**F:** leadership; preparedness arrangements; external networks.	High across dimensions; leadership and culture highest and change ready lowest, descriptively	The overall mean BRT score was 4.2 (standard deviation [SD] 0.41) on a five-point scale. Leadership and culture averaged 4.3 (SD 0.21), networks and relationships 4.2 (SD 0.65), and change ready 4.0 (SD 0.33); planning resilience averaged 4.2 (SD 0.44) and adaptive capacity 4.2 (SD 0.42). These descriptive ratings indicated high perceived resilience across dimensions, while qualitative accounts showed greater uncertainty about preparedness for prolonged future crises. [*No inferential comparisons or associations were tested*].
**Soleimani 2025, 2026**	F: digital technologies; preparedness arrangements. C: reconfigure.	CRM resources → stronger capabilities → stronger risk management; risk and crisis management → higher resilience	CRM resources were positively associated with organizational capabilities, which predicted risk management. Risk management (β=0.337) and crisis management (β=0.619–0.691) were positively associated with resilience. The later analysis (2026) found that organizational capabilities fully mediated CRM–risk management, while risk and crisis management showed partial mediation in the pathways to resilience.
**Tongtong 2024**	**F:** leadership; digital technologies.	Strategy, leadership, digital technology, and risk control → higher resilience; strategy → no association with risk control	Risk-control capability was positively associated with hospital resilience (β = 0.280, p < 0.001). Strategy formulation (β = 0.174, p = 0.030), leadership (β = 0.163, p = 0.049), and digital-technology capability (β = 0.141, p = 0.046) also had positive direct associations. Leadership and digital technology were positively associated with risk control, whereas strategy formulation was not. Risk control partially mediated the leadership–resilience and digital-technology–resilience associations but not the strategy–resilience association. The model explained 32.6% of hospital-resilience variance.
**Tongtong 2025**	**F:** digital technologies.	Synergy and innovation → higher resilience; compatibility and security → no significant association	Digital-technology synergy was positively associated with hospital resilience (β = 0.247, t = 2.969, p = 0.002), and innovation had the largest positive association (β = 0.343, t = 3.744, p < 0.001). Compatibility was not significantly associated with resilience (β = 0.158, t = 1.404, p = 0.080), and security was also nonsignificant (β = −0.018, t = 0.165, p = 0.435). The model explained 44.1% of the variance in resilience (R^2^ = 0.441) and had reported predictive relevance (Q^2^ = 0.209).
**Tortorella 2021**	**F:** digital technologies. **C:** anticipate; learn.	Four consistently high impact; two consistently low impact; others varied by resilience ability	Remote consultations and real-time care planning, digital non-invasive care, interconnected medical-emergency support, and collaborative patient-data platforms were classified as high impact across monitoring, anticipating, responding, and learning. Medical-device traceability and computer-assisted design of customized medical devices were consistently low impact; the remaining technologies had ability-specific impacts.
**Yun 2025**	**F:** preparedness arrangements; workforce resources.	Mixed; plans and response teams were common, but operational readiness and resources were limited	Although 85.7% reported a business-continuity plan, 57.9% considered its implementation insufficient. Only 9.5% had an infectious-disease response team before COVID-19; 71.4% formed one during the first wave and 19.0% during the second. Manpower management was the greatest cohort-isolation difficulty (mean 4.33, standard deviation 0.73), while communication was weakest for staffing and financial support.

**Note:** F = organizational factor; C = resilience capability. The factor and capability terms listed for each study reflect the specific component(s) supported by that study and map to the six higher-order factor domains and five higher-order capability domains used in the synthesis.

**Table 3:** Integrated synthesis of factors associated with organizational resilience in healthcare organizations

Factor domain	Principal factors	Evidence across qualitative and quantitative studies
Leadership, governance, and decision authority	• Decision authority and accountability • Leadership structures and participation Strategic–frontline coordination • Central direction with local autonomy	Qualitative evidence linked clear authority, participation and strategic–frontline coordination with timely action; ambiguous or contradictory authority constrained trust and learning. Quantitative findings generally favored self-organization and leadership, including pathways through well-being and risk control, although some governance and network paths were null or mixed.
Workforce resources, well-being, and organizational culture	• Workforce availability and staffing capacity • Clinical and operational expertise • Cross-training and flexible staffing arrangements • Staff well-being support, teamwork, and trust	Qualitative evidence linked flexible deployment, competence, teamwork and support with continuity; shortages, illness, fatigue and repeated redeployment constrained sustained response. Quantitative findings generally favored human-resource management, occupational safety, engagement, well-being and staffing intensity, while some skills effects were indirect.
Information systems, communication, and digital technologies	• Communication channels and information quality • Internal and external information integration • Digital and knowledge-management infrastructure • Technology fit, implementation, and trust	Qualitative evidence linked timely communication and usable digital systems with coordination and adaptation; fragmented or poorly documented information constrained resilience. Quantitative findings favored external information integration, selected digital innovations and AI-enabled knowledge management, while several skills, internal-integration, compatibility and security paths were null or context-dependent.
Preparedness arrangements, organizational structures, and coordination arrangements	• Preparedness and business-continuity plans • Incident-command and crisis structures • Protocols, simulations, and training • Role structures and coordination mechanisms	Qualitative evidence showed that plans and crisis structures supported initial response but required revision as disruptions evolved. Quantitative and mixed-methods findings favored formal and informal practices, with stronger associations for informal practices in one study; resource reconfiguration was favorable, while anticipation and implementation gaps remained.
Material, financial, and infrastructural resources	• Physical space and surge capacity • Supplies, equipment, and infrastructure • Redundant and substitutable capacity • Flexible financing and continued resource access	Qualitative evidence showed that shortages constrained action, while redundancy, adaptable space and continued external support extended capacity. Quantitative findings were mixed: staffing intensity and temporary relief were favorable in specific settings, many measured hospital characteristics were unrelated to recovery, and caseload strain predicted worse outcomes.
External networks, community relationships, and policy support	• Interorganizational partnerships and networks " Community relationships and trust " Government and public-health support • Policy, regulation, and external financing	Qualitative evidence linked partnerships, regional coordination, community relationships and government support with access to staff, information and supplies; fragmented or misaligned arrangements constrained action. Quantitative findings favored external information integration and temporary policy or financial support, but regulation, network and regional pathways were mixed.

## Research in Context

### What is already known about the topic?

Previous reviews have identified leadership, workforce capacity, communication, preparedness, resources, and external relationships as recurring components of healthcare organizational resilience. However, definitions and measures often combine organizational factors, resilience capabilities, and outcomes, making studies difficult to compare. Most evidence concerns hospitals and major disruptions.

### What does this study add to the literature?

This review integrates 47 reports representing 45 studies into an evidence-informed framework distinguishing six factor domains, five capabilities, four outcome groups, and contextual conditions. It also shows that factor–resilience relationships vary across contexts and that short-term service continuity can coexist with workforce overextension, deferred care, temporary financial support, or resource depletion.

### What are the policy implications?

Policymakers and healthcare leaders should assess preparedness beyond the existence of plans and monitor service continuity, safety and quality, workforce well-being, deferred care, recovery time, and organizational viability during and after disruption. Assessments should also examine whether organizations can mobilize and adapt resources as demands change; the framework can structure such assessments but has not been validated as an assessment instrument.

## Abbreviations

3SLS: Three-stage least squares
AGENAS: Agenzia Nazionale per i Servizi Sanitari Regionali
AI: Artificial intelligence
AI-KMS: Artificial intelligence integration within knowledge-management systems
AIDS: Acquired immunodeficiency syndrome
BRT: Benchmark Resilience Tool
CHVNGE: Centro Hospitalar de Vila Nova de Gaia/Espinho
CI: Confidence interval
CMIN: Centro Materno Infantil do Norte
COVID-19: Coronavirus disease 2019
CRH: Clinical Resource Hub
CRM: Customer relationship management
HERO-36: Healthcare Emergency Response Optimization 36-item survey
HIV: Human immunodeficiency virus
HOR: Hospital organizational resilience
HRI: Hospital Resilience Index
HRM: Human resource management
ICU: Intensive care unit
ICT: Information and communication technology
IT: Information technology
JBI: Joanna Briggs Institute
KMS: Knowledge-management systems
NHS: National Health Service
OHS: Occupational health and safety
OLS: Ordinary least squares
PLS-SEM: Partial least squares structural equation modeling
PPE: Personal protective equipment
PRF: Provider Relief Fund
PRISMA: Preferred Reporting Items for Systematic Reviews and Meta-Analyses
RAG: Resilience Analysis Grid
SD: Standard deviation
SE: Staff engagement
SHRM: Strategic human resource management
SOP: Standard operating procedure
SR: Staff resilience
TUTH: Tribhuvan University Teaching Hospital
VHA: Veterans Health Administration
WHO: World Health Organization
